# The Breast Cancer Single-Cell Atlas: Defining cellular heterogeneity within model cell lines and primary tumors to inform disease subtype, stemness, and treatment options

**DOI:** 10.1007/s13402-022-00765-7

**Published:** 2023-01-04

**Authors:** Arpit Dave, Daniel Charytonowicz, Nancy J. Francoeur, Michael Beaumont, Kristin Beaumont, Hank Schmidt, Tizita Zeleke, Jose Silva, Robert Sebra

**Affiliations:** 1 Department of Genetics & Genomic Sciences, Icahn School of Medicine at Mount Sinai, 1425 Madison Ave - Icahn (East) Building, Floor 14, Room 14-20E, New York, NY 10029 USA; 2 Icahn Genomics Institute, Icahn School of Medicine at Mount Sinai, New York, NY 10029 USA; 3 GlaxoSmithKline, Collegeville, PA 19426 USA; 4 Department of Pathology, Icahn School of Medicine at Mount Sinai Hospital, New York, NY 10029 USA; 5 Black Family Stem Cell Institute, Icahn School of Medicine at Mount Sinai, New York, NY 10029 USA; 6 Center for Advanced Genomics Technology, Icahn School of Medicine at Mount Sinai, New York, NY 10029 USA; 7 Pacific Biosciences, CA Menlo Park, USA

**Keywords:** Breast Cancer, scRNAseq, Cell Lines, Stemness Scoring, Disease Subtyping, Therapeutic Prediction

## Abstract

**Purpose:**

Breast Cancer (BC) is the most diagnosed cancer in women; however, through significant research, relative survival rates have significantly improved. Despite progress, there remains a gap in our understanding of BC subtypes and personalized treatments. This manuscript characterized cellular heterogeneity in BC cell lines through scRNAseq to resolve variability in subtyping, disease modeling potential, and therapeutic targeting predictions.

**Methods:**

We generated a Breast Cancer Single-Cell Cell Line Atlas (BSCLA) to help inform future BC research. We sequenced over 36,195 cells composed of 13 cell lines spanning the spectrum of clinical BC subtypes and leveraged publicly available data comprising 39,214 cells from 26 primary tumors.

**Results:**

Unsupervised clustering identified 49 subpopulations within the cell line dataset. We resolve ambiguity in subtype annotation comparing expression of Estrogen Receptor, Progesterone Receptor, and Human Epidermal Growth Factor Receptor 2 genes. Gene correlations with disease subtype highlighted *S100A7* and *MUCL1* overexpression in HER2 + cells as possible cell motility and localization drivers. We also present genes driving populational drifts to generate novel gene vectors characterizing each subpopulation. A global Cancer Stem Cell (CSC) scoring vector was used to identify stemness potential for subpopulations and model multi-potency. Finally, we overlay the BSCLA dataset with FDA-approved targets to identify to predict the efficacy of subpopulation-specific therapies.

**Conclusion:**

The BSCLA defines the heterogeneity within BC cell lines, enhancing our overall understanding of BC cellular diversity to guide future BC research, including model cell line selection, unintended sample source effects, stemness factors between cell lines, and cell type-specific treatment response.

**Supplementary Information:**

The online version contains supplementary material available at 10.1007/s13402-022-00765-7.

## Background

Breast Cancer (BC) is a blanket term used to describe any neoplastic growth in the breast and its neighboring tissues, with 13% of women developing invasive breast cancer in the United States in their lifetime [1]. While we observe up to a 1% decrease in death rates for specific patient populations, estimates indicate over 43,000 deaths per year in the US attributed to breast cancer. However, through improved disease characterization and patient disease modeling, a precision medicine approach to treatment can reduce the burden of breast cancer recurrence and mortality in today’s healthcare system [2].

**Fig. 1:**
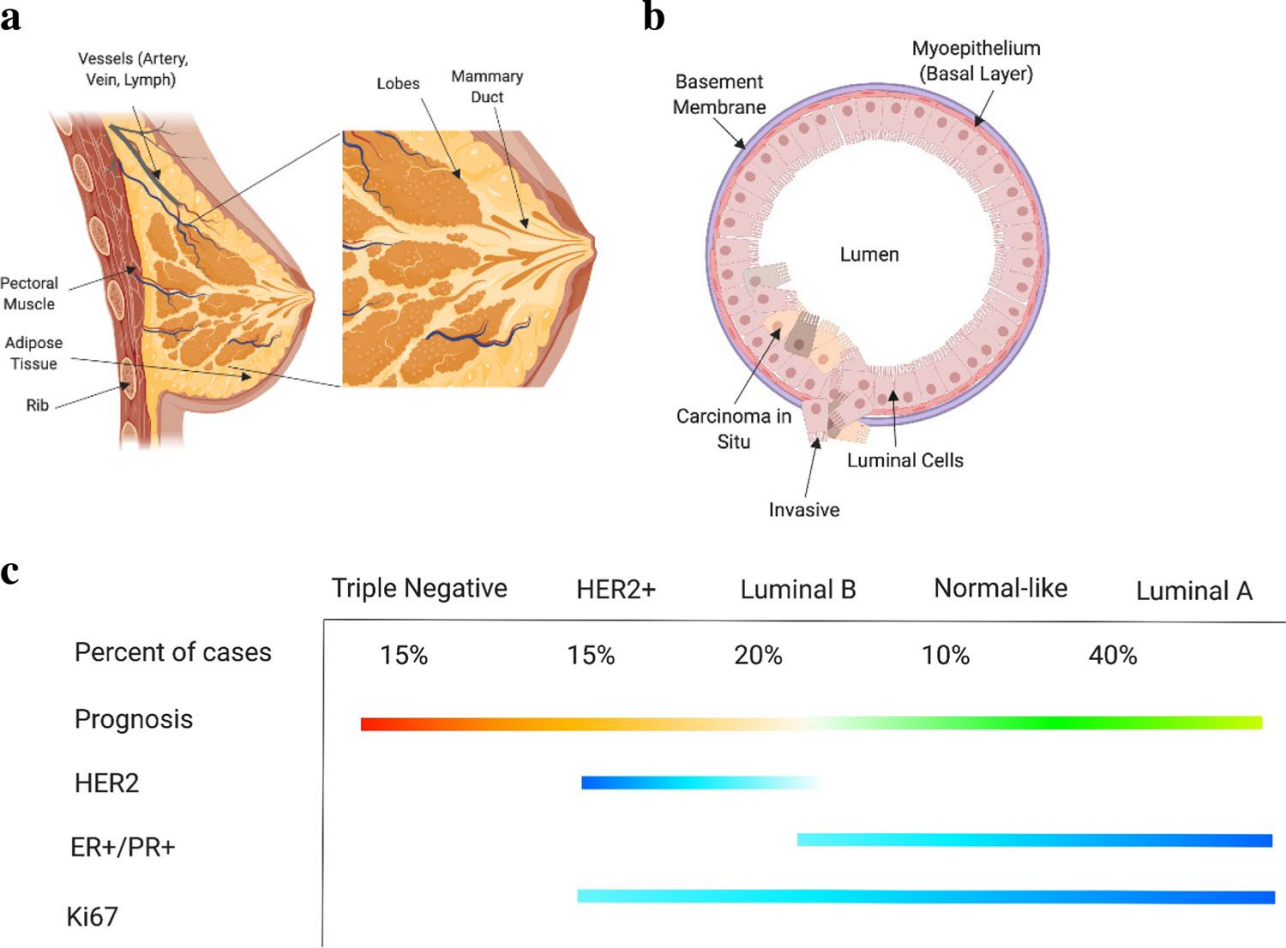
Background overview of breast cancer anatomy and disease subtyping. **a** Whole tissue breast anatomy defining key functional regions including mammary duct and lobes. **b** Breast cellular anatomy defining cell types and duct structure. **c** Subtype breakdown of breast cancer by common protein and cell surface marker expression. Each of these illustrations were generated on Biorender.com

To better characterize the heterogeneity of breast cancer, disease classification is contingent on four factors: histological type, tumor grade, stage, and molecular expression levels of specific proteins [3]. Histological classification apportions BC to either carcinoma in situ (CIS) or invasive carcinomas, which are further stratified by cellular origin within functional sub-compartments of the tissue. For example, the CIS classification is composed of both ductal carcinomas (DCIS) (Fig. 1a) and lobular carcinomas (LCIS), where ductal and lobular define the regions of disease origin within the breast. The lobules in a gland are responsible for milk production, which are then delivered to the skin surface via the ducts (Fig. 1b). The Elston & Ellis grading system is a classification system for BC based on tumor cell differentiation and metastatic potential [4], where Grade 1 is the most differentiated and slow-growing. Increasing grades represent decreasing differentiation and higher proliferation. In conjunction with grading, there is a staging system with five standard classifications dependent on disease localization [5]. Stage 0 (DCIS) represents in situ disease with an estimated 100% survival rate. Increased staging denotes larger tumor sizes, metastases to lymph nodes and adjacent organs, and decreased survival rate. Stage 4 BC has a 24.5% five-year survival rate [6]. Expression level classification of BC is a critical tool commonly utilized for selecting the most appropriate treatment strategies. Figure 1c illustrates expected marker expression across the distinct subtypes: Triple Negative (TNBC), HER2 + , Luminal B, and Luminal A. These molecular subtypes are generated by expression of the Estrogen Receptor (ER), Progesterone Receptor (PR), and Human Epidermal Growth Factor Receptor 2 (HER2). Each of the positively expressed receptors in the subtypes has been studied as targeting methods for therapies. TNBC has no positive surface expression of these canonical receptors, thereby reducing potential targeting methods for therapies. This, in conjunction with TNBC representing the most heterogeneous molecular subtype, primarily drives poorer patient outcomes. Based on the classification criteria complexity, disease characterization varies between labs. This is primarily due to a lack of higher resolution data (reliance on immunohistochemistry) characterizing disease. Furthermore, the notations utilized by researchers and healthcare professionals are often inconsistent, leading to unintentional complexity in disease subtyping and classification.

The high prevalence of BC has fueled research into promoting healthier lifestyle choices, refining diagnostic measures, and expanding disease prevention and treatment options to improve patient outcomes. With over 500 ongoing BC clinical trials, as of 2021, aimed at addressing the prevalence of disease, 183 of which are exploring targeted agents, there is a pressing need for systemic testing of these candidate therapies [7]. A significant driving force in biomedical research is the availability of technology to define patient disease populations. With growing next-generation sequencing (NGS) and single-cell sequencing assays, there has been a trend towards replacing blanket therapies with patient-specific therapeutics [8]. For this to be successful, there needs to be improved identification of cellular heterogeneity comprising patient tissue as well as in model systems used to investigate these disease subtypes. As our understanding of the complexity of cancer evolves, the resolution of data needed to provide an accurate framework for therapeutic targets should approach single-cell resolution.

The need for higher resolution data is further highlighted by the confounding of cell line subtyping across publications [9]. Extensive bulk analysis has paved a strong foundation for inter-tumor heterogeneity identification and treatment. However, the granularity within a tumor is often left unaddressed. Due to unique levels of marker thresholds for proteins or genes, binning model lines to subtypes vary across institutions and experiments. This is further confounded by discordant ER, PR, and HER2 + status between multiple samples from a patient attributed to reasons including a change in cancer biology between sample sites and timepoints, sampling error, and accuracy and reproducibility of the receptor assays [10]. Cell lines like MDAMB453 are particularly confounded where discrepancies in protein and gene expression of HER2/*ERBB2* yield both TNBC and HER2 + subtype annotations [11]. This level of heterogeneity is variable across cell lines. Therefore, deeper scRNAseq analysis across model lines can elucidate attributes such as heterogeneity, stemness, cell line markers, functional cluster prediction, and therapeutic prediction that may significantly impact the marriage between cell line selection and study outcomes.

The first cultured cancer cell lines in the 1950s represented a landmark in cancer biology. Since then, the repertoire of cancer lines has expanded to reach over a thousand lines for almost all known tumor types. Specifically, cancer research has relied on model cell lines as a pretest across various experimental processes such as gauging response to therapy [12]. The importance of these lines as experimental models cannot be overstated. As an illustrative example, the breast cancer cell line MCF-7 has functioned as established benchmarks across the field, with over 100,000 publications testing this individual line [13]. Thus, our current ability to study the process of transformation and experimentally interrogate therapeutic avenues cannot be understood without these experimental models. Precise characterization of cell lines is critical to utilize their maximum potential. During the last decade, the growth of NGS has supported the characterization of cell lines at a molecular level [14].

Using bulk sequencing, gene expression profiling has been widely adopted and incorporated for tumor samples and model cell lines. In part due to the long-term adoption of these technologies, there is an abundance of available analysis pipelines and predictive tools for this data type. For example, Schafer et al. developed a pipeline to leverage microarray data for quick on-site prediction of recurrence through hormone receptor status [12]. This tool, and many others, provided clinicians and researchers with necessary validation and prediction pipelines based on the broadly available bulk RNAseq datasets. However, intratumoral heterogeneity is well recognized as the main problem that compromises the response to anticancer therapy. The growth of single-cell sequencing technologies allows us to study this phenomenon with unprecedented resolution. Remarkably, heterogeneity has also been previously identified in cultured cells and cells with different features such as morphology [15], ploidy [16], and gene expression [17]. Despite this, the characterization of cell lines using single-cell technologies is still in its infancy. While there has been rapid adoption of these technologies to characterize patient tumor biopsies, there remains a void in publicly available single-cell data to characterize widely used model cell lines [14].

To date, there has not been a comprehensive single-cell dataset across all breast cancer cell lines to elucidate this confounding heterogeneity and better parse which lines are most appropriate for specific disease subtypes and functional testing. Here we present the first BSCLA dataset and outline a pipeline for highly resolved characterization to determine intra-cell line heterogeneity and provide recommendations for cell line selection through factors including heterogeneity scoring, stemness features, and subcluster defining gene vectors. The cell lines processed for scRNAseq are consolidated in Table 1 with comprehensive data metrics. We overlay this analysis with primary tumor epithelial and mesenchymal cells to highlight the value of model systems when chosen and incorporated in an informed manner.

**Table 1:** Sequencing quality control metrics for all the BC lines processed

Cell Line	Cell Count	Median Genes per Cell	Valid Barcodes (%)
SKBr3	518	3208	97.7
MDA-MB-361	1422	4704	97.1
BT-474	4210	4162	97.8
MDA-MB-453	4837	1705	96.8
MCF-7 – Sample 1 – Run 1	2340	3981	97.2
MCF-7 – Sample 1 – Run 2	2683	2707	98.0
MCF-7 – Sample 2	5217	3109	98.2
T47D	5539	3908	98.1
MDA-MB-468	1986	4762	97.4
SUM190	1445	3636	97.1
HCC1954	1579	1066	96.7
SUM149	1517	5305	97.2
BT-549	3425	5393	98.0
MDA-MB-436	4521	2827	96.8
MDA-MB-231	991	4077	97.2

This table summarizes sequencing quality control metrics for each sample processed through scRNAseq. Cell count represents the total yield of bead barcodes prior to data filtering. Median genes per cell is a representation of data sequencing depth and cell activity. Valid barcodes are the percentage of barcodes successfully mapping to the human genome

## Methods

### Cell line culture, harvesting, and imaging protocol

Breast cancer cell lines were thawed from frozen aliquots and cultured in CytoOne T25 flasks (USA Scientific) with culture media composition dependent on ATCC guidelines. Typical media recipes included DMEM/DMEM/F-12 media (Thermo Fisher), 5% FBS (Sigma-Aldrich), 1% Pen-Strep (Gibco), supplemented with 10 µg/ml Insulin (Sigma-Aldrich), and 5 ng/ml Endothelial Growth Factor (Thermo Fisher) depending on the cell line. Cells were incubated at 37 °C in a humidified 5% CO_2_ atmosphere. Upon 80% confluency, the sample was passaged following the recommended sub-culturing protocol for adherent cells. All cell lines were cultured for a maximum of five passages from the original ATCC sample collection to limit divergence from the original reference strain.

### Single-cell RNA sequencing and library prep

For single-cell RNA sequencing, cell lines were collected before passage 5 and suspended in 1X PBS (calcium and magnesium-free) containing 0.04% weight/volume BSA (400 µg/ml) at 1 × 10^6^ cells/mL. Cells were processed according to Chromium 3’ Gene Expression V3 Kit (10X Genomics) using manufacturer’s guidelines followed by sequencing on an S1 NovaSeq chip (Illumina Inc.). Quality check of cDNA was done with Qubit 3 (Fisher Scientific) and High-sensitivity 2100 Bioanalyzer (Agilent). The 10X Cell Ranger software v3.1.0 was used to process the BAM file from sequencing. This outputs a read counts matrix that we used for downstream analyses using Seurat, a customizable R-Studio Package for scSEQ analysis [18]. At least ~ 20,000 2 × 150 bp reads per cell were generated for each of the 36,195 cells, with an average 97% mapping rate.

### Post sequence data processing

10X Genomics Cell Ranger software v3.1.0 was used to process files from each sample. This generated a counts matrix file [19]. FASTQ files were generated from demultiplexed raw base call (BCL) files through the Cellranger mkfastq pipeline. The Cellranger count pipeline was applied to FASTQs to perform alignment against GRCh38 human reference build, filtering, barcode counting, and UMI counting. The feature-barcode matrices were analyzed through a series of open-source R platforms, including Seurat (Satija Lab) and ClusterProfiler (He Lab) [20]. The counts matrix is a fundamental unit of scRNAseq where column barcodes correspond to cell identities and rows are filled by gene names. The matrix values represent detected gene expression with the representative individual cell barcode. The counts matrix is a fundamental unit of scRNAseq where column barcodes correspond to cell identities and rows are filled by gene names. The matrix values represent detected gene expression with the representative individual cell barcode. Each cell line dataset is independently filtered for nFeature_RNA, nCount_RNA, and mitochondrial gene percentage. By filtering these values, we can computationally reduce the composition of dead cells, dying cells, and duplicates within our dataset. This multimodal expression has been previously described by Bacher et al. [21]. When selecting a minimum number of unique features(nFeatures), the range between samples is from 1,600 to 2,000. Similarly, when selecting for a maximum number of total features (nCount), the range is from 40,000 to 65,000. Lastly, for percent mitochondrial the range for maximum percentage of mitochondrial genes (percent.mito) varies from 16 to 25%. Each filtered cell line dataset is then merged to create global dataset that can be analyzed as one unit. The Seurat object is processed similar to previous methods with normalization done using the LogNormalize method and a scale factor of 10,000. The FindVariableFeatures() is set to select using VST and for a number of features at 2,000. Cell cycle scoring measures s phase and g2m phase gene using the CellCycleScoring() function. Scaling is done to regress for percent mitochondrial, nCount_RNA, s phase score, and the G2M phase score using the ScaleData() function. FindNeighbors() is run to calculate distance relationships with 20 dimensions included in the analysis, determined from an elbowplot. Clustering of the tissue dataset is set at a resolution of 0.8 and done with the FindClusters() function. This is followed by unsupervised clustering of the tissue dataset with 13 dimensions included in the analysis.

### Published data import

Single-cell RNA sequencing data files were downloaded from The Broad Institute Single Cell portal. The downloaded dataset is pulled using Seurat's Read10X() function and then converted into a Seurat object using CreateSeuratObject(). The data is filtered for nFeature_RNA less than 8,000, nCount_RNA greater than 1,000, and mitochondrial genes less than 8 percent. This integrated dataset is then processed with the steps done earlier to reduce batch effects including NormalizeData(), FindVariableFeatures(), CellCycleScoring(), and ScaleData(). The last preprocessing stage is generating UMAP for the primary tumor cells using 13 dimensions. Using the canonical markers *EPCAM, PDGFRB*, *MKI67*, and *CD68* identified and leveraged by Wu et al., epithelial and mesenchymal cells were selected to create a subset Seurat object. This subset is merged with the preprocessed cell line atlas dataset for all analysis conducted. The merged atlas containing primary tumor epithelial cells, primary tumor mesenchymal cells, and breast cancer cell line data is re-normalized within the Merge() Seurat function. This merged dataset is scaled to reduce technical variability between sample types. Harmony data integration was leveraged when comparing independent HER2 cell lines with HER2 expressing patient tumor data [22]. This integration used 16 dimensions identified from an elbow plot and a resolution of 0.2 for reclustering.

### Resolving subpopulation heterogeneity within cell lines

PCA is useful for fast and linear dimensionality reduction, however with increasingly complex data affiliated with scRNASeq, UMAP is another preferred network analysis tool that preserves global structure, distance correlations, and continuity of cell states. Cell clustering was performed with the FindClusters() Seurat function. UMAP dimensionality reduction was done with the RunUMAP() Seurat function. Using the filtered, normalized, and scaled dataset, cell line clustering provides increased resolution to gene expression and clonal population differences. We can further highlight potential functional clustering by investigating differential gene expression between clusters within this dataset. This analysis averages data points across an identified subpopulation to extrapolate distance relationships. We explored all markers expressed in each cluster and sorted by the difference of pct.1 and pct.2, representing the percent of cells in a specific cluster expressing a gene and the percent of cells outside that cluster expressing that gene, respectively. We have identified that genes with difference values greater than 0.5 are responsible for providing the most direct representation of individual cluster states and that these genes, in most cases, parallel the most significant differentially expressed gene (DEG) with regards to p-value and Avg-logFC. Through sorting by this difference value, we derive gene expression sets increasingly specific to the subpopulation of interest.

### Characterizing individual cell lines and their gene signatures

Using the FindMarkers() function, we identify the differentially expressed genes for individual subpopulations compared to the entire global dataset or the local individual sample set. This function utilizes a Wilcoxon Rank Sum Test to identify differential genes between two populations. The output of FindMarkers() is then filtered for strict avg_logFC (> 0.8) and difference (> 0.5) values. In parallel, the FindMarkers() function is run across nodes identified from the BuildClusterTree() function for identifying observed population divergence driving genes.

### Fluorescent protein expression assay and analysis

The MDA-MB-453 cell line was cultured to passage 2. Cells were diluted to a concentration of 1e6/ml in PBS (Thermo Fisher) and incubated with Anti-ERBB2 Affibody Molecule with FITC conjugation (Abcam) at a 1/100 dilution. A 30-min incubation was followed by centrifuge at 100 g for 6 min with the Centrifuge 5702 (Eppendorf). The supernatant was removed, and cells were diluted with the PBS buffer. The EVOS M7000 (Thermo Fisher) was used for microscopy imaging, cell counting, and fluorescence intensity measurements. The focus was automatically calibrated by the brightfield channel. The fluorescent images were analyzed using the EVOS analysis software system (Thermo Fisher).

The Beacon instrument (Berkley Lights) was leveraged for high throughput cellular organization and imaging. A microfluidic chip was used that allows isolated single-cell imaging and functional experimentation. After ERBB2 antibody incubation and wash, cells were immediately imported into the microfluidic chip containing 3500 isolated nanopens. Using OptoElectroPositioning (OEP), individual cells are manipulated and moved from the flow channel into the nanopens. Following import, the cells are cultured in their regular media (L-15 with 10% FBS). The entire chip is imaged in both brightfield and FITC imaging channels. The chip comprises 22 Fields of View (FOV), each of which is an image per channel. To analyze the data across the 3500 nanopens in the 22 FOVs in both imaging channels, the files are exported and processed through a custom-engineered MATLAB image analysis script. In parallel, MDA-MD-453 cells are imaged using traditional microscopy on an EVOS m7000 (Thermo Fisher) with the GFP channel, Images from the EVOS microscopy are investigated using Image Analyzer (Thermo Fisher). Within the MATLAB script, cell locations are identified using a Hough Transform circle detection algorithm. Detected cells are then filtered by cell brightness not passing the traditional threshold of live cells, masking due to chip background interference, and location filtering due to cell import and localization near the top of a nanopen. Locations of each detected cell are saved in a matrix, and brightness measurements are automatically pulled at those pixel locations for both imaging channels. FITC brightness is normalized by dividing expression recording by mean expression across all detected cells. To determine positive expression, cells were grouped using a k-means clustering algorithm based on the ERBB2 expression vector, using a k score of 2.

### Sub-clustering across the global BSCLA dataset

To organize each sample set, we generated a Seurat object representative of each cell line processed at a given time point. The Seurat object is named by cell line, sample source, and run number. For example, MCF-7s1r2 represents the MCF-7 cell line from sample source 1, which was run for a second time. For samples that we only processed once and without multiple time points or sources, we bypass these features on the nomenclature and are instead denoted by their cell line name.

We first filter each Seurat object to account for variation in cell loading concentrations, cell viability, and processing. As a natural byproduct of the current microfluidic system used for single-cell isolation, there is a percentage of Gel beads in emulsion (GEM) that contain multiple cells or dying cells which, we can computationally remove from the analysis [23]. We filtered GEM barcodes with high percent mitochondrial genes (dead cells), high total RNA (multiplets), and low unique RNA (empty GEM). The thresholds of cutoff variate between samples, and therefore, rather than employing global absolute value filters, we identify extreme expression cutoffs by percent calculated for individual samples. For example, in each cell line, the threshold for high percentage mitochondrial gene expression is determined by bimodality of expression, fluctuating between 4.3–12.1 percent mitochondrial genes in a cell line, depending on the sample.

To minimize technical variability in our dataset across samples, NormalizeData() was run across each cell line to reduce biasing by cell total transcription. This is followed by the ScaleData() function, which shifts mean expression for every gene across all cells to 0 and standardizes the variance of each gene to 1. This process is standard in scRNA-seq pre-processing and is described extensively in literature. Once the Seurat objects have been independently filtered, we merge the objects for population analysis and comparison. These preprocessing steps are necessary to minimize outstanding cell populations not representative of healthy cells in culture. The cells are then processed for unsupervised clustering, which groups cells based on distance relationships in the global dataset. These clustered populations yield subpopulations that can be investigated for functional predictions and define heterogeneity across and within the cell lines.

### Entropy scoring pipeline for stem-like population identification

This method of entropy scoring for stemness analysis is discussed earlier in Panebianco et al. [19]. The degree of “stemness” was estimated for all cells using the Shannon entropy transcriptional scoring method first proposed by *Tessendorf* et a. 2017 [24]. In brief, it has been shown that cells with increased differentiation potency (i.e., stemness) exhibit higher signaling entropy as measured on gene expression patterns overlaid across protein–protein interaction (PPI) networks. Stem-like cells, in general, exhibit more diffuse expression of gene signaling pathways, whereby during the process of differentiation, cell-type-specific pathways remain active while non-specific pathways are progressively pruned away and deactivated. This general observation can be quantified by calculating signal entropy of a Markov-chain created by integrating cell-specific gene expression patterns with a fully connected PPI matrix, and in turn results in stem-like cells exhibiting high signaling entropy, while differentiated cells exhibit low signaling entropy.

The calculation of entropy scores is, however, a computationally intensive procedure involving several large matrix operations. To optimize this calculation and enable its use for larger datasets such as our breast cancer cell line atlas, it was necessary to re-implement the entropy scoring algorithm in Tensorflow 2.0, adding support for rapid batch processing and GPU acceleration. Entropy calculation for breast cancer cell lines was performed using our optimized implementation on a Google Cloud VM instance consisting of a 4-core Intel Xeon E5-2630 2.3GhZ CPU running on an NVIDIA V100 SMX2 GPU with 25 GB RAM.

Following score calculations, Spearman correlations were computed for all genes and individual cells, with Bonferroni correction for multiple statistical testing. Genes shown to have a significant positive correlation to entropy score can be interpreted as being overexpressed in high-entropy, stem-like cells. Conversely, genes with a negative correlation to entropy are considered overexpressed in low-entropy (differentiated) cell populations. Gene sets were then interrogated with orthogonal pseudo time calculation approaches to discern markers of stem-like states in breast cancer cell lines.

## Results

### scRNAseq reveals heterogeneous populations across BSCLA cell lines

**Fig. 2:**
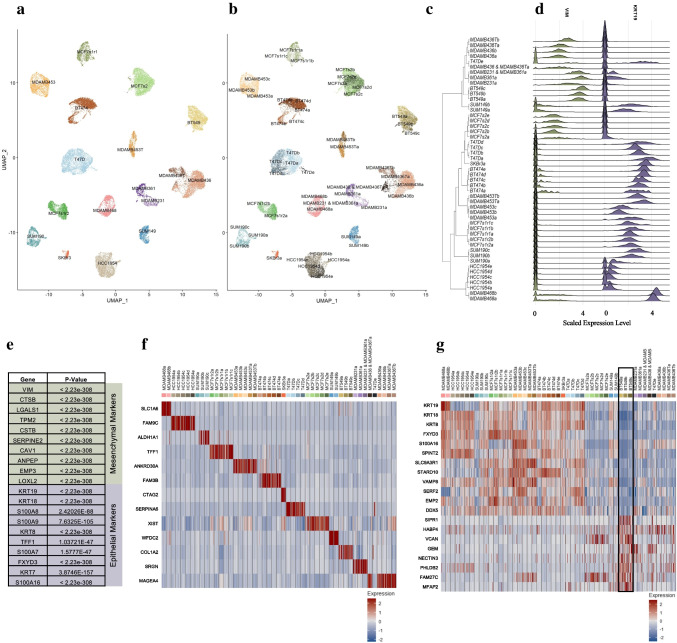
Cluster analysis of global dataset for population gene vector identification. **a** UMAP plot annotating cell populations by the 13 diverse cell lines of origin and replicates. **b** UMAP plot annotating cell populations by the 49 unsupervised cluster annotations. **c** Phylogenetic tree organizing subclusters across global population by bulk gene comparison and define distance relationships between groups. **d** Deriving differential genes driving nodal splits in the phylogenetic tree. Vimentin and Cytokeratin 19 are key genes in the first global nodal split differentiating epithelial cells from mesenchymal. **e** Table outlining top 10 marker genes for epithelial vs mesenchymal split, identified by Avg_log2FC value. **f** Heatmap expressing individual genes with significant expression in their subpopulation. **g** Heatmap highlighting the vector of genes differential to BT-549. Reproduced for each cell line. Positive and negative gene expression function to isolate cell lines of interest successfully

13 unique breast cancer cell lines were processed for scRNAseq, with additional sample replicates for the MCF7 line, Fig. 2a. Unsupervised graph-based clustering resolved 49 unique clusters based on nearest neighbor approximations**,** with clusters and annotated subclusters outlined on the UMAP plot in Fig. 2b. We observe cell line of origin as the primary differentiator between clusters. Therefore, subcluster nomenclature is annotated by cell line name, sample-ID, run number, and cluster-ID. For example, in Fig. 2c, all UMAP clusters are shown by cell line names such as MCF-7s2 (MCF-7 cell line, sample ID 2). In Fig. 2b, this representative cell line is compartmentalized further into 5 subclusters: MCF-7s2a, MCF-7s2b, MCF-7s2c, MCF-7s2d, and MCF-7s2e (Supplementary Table 1).

To analyze this merged global dataset of all cells across lines, a phylogenetic tree was generated using BuildClusterTree to compare subclusters on a populational level (Fig. 2c). BuildClusterTree is a function provided by the Seurat scRNAseq analysis pipeline (see Section 2) that allows the prediction of forced distance relationships between cell lines as well as subclusters [18]. Phylogenetic trees were generated by comparing distances between computed average cells to define each end subcluster. Each split in the tree is defined by a combination of differentially expressed genes driving the differences in cell type. As the analysis progresses into higher resolution branch splits, individual subclusters and the historical gene differences to arrive at each end population are identified. The vector of gene markers defining nodal splits are then compiled to represent each subcluster. When comparing these markers with literature, there is high concordance with mesenchymal and epithelial gene markers such as *VIM* and *KRT19*, respectively (Fig. 2d). After applying stringent filtering criteria, including an Avg-LogFC value > 0.8 and a difference value > 0.8, comprehensive gene vector sets are generated for each population. The difference value represents the disparity between the fraction of cells in the subclusters that express a gene and the fraction of cells outside of the population that express that gene. Avg-LogFC represents log-fold differences in expression between populations. Through combining Avg-LogFC and difference filter parameters, the end gene vectors factor intensity and frequency. The high sample count in scRNAseq provides increased power in each gene comparison resulting in significance below 0.05 for all genes of interest identified in the analyses below. Coordinated analysis across each subpopulation generates gene vectors for each subpopulation. For example, Fig. 2e highlights top gene expression differences, sorted by Avg-LogFC, driving the first nodal split, representing epithelial and mesenchymal cells. While any of these gene markers, including *VIM* and *KRT19*, are known for the defining cell type, there is also an observed novel gene set for both populations. For example, *MT1E* is the most significant differentially expressed gene with an absolute Avg-LogFC greater than two between the mesenchymal and epithelial populations. *MT1E* has been implicated in migration and invasion within cancer cell lines, and its concordance with mesenchymal cell populations is further supported by this cell type characterized with higher invasiveness [25]. Significant gene expression of *FXYD3* is observed in the epithelial population. *FXYD3* has been linked to overexpression in many cancer types and correlated with fertility frequency, thereby linking the high concordance of this gene with breast and endometrial cancers [26].

This nodal analysis was then extended across the nodes defining each population split. This allows us to create gene vectors for each cell line and subclusters in our population as outlined by the phylogenetic tree. Supplementary Table 2 is a comprehensive list of gene markers specific to each BSCLA line p_Value, Avg-LogFC value, and difference values for significance. Figure 2f is a heatmap of a key differentially expressed gene for each cell line, highlighting the specificity of gene expression vectors across the population. While not every gene in the generated gene set is ubiquitously specific to a subpopulation, this signifies the complexity of the gene vectors to represent a cell population successfully. To highlight this, genes composing the vector set for BT-549 (*highlighted within the annotated black box*) were chosen, shown in Fig. 2g, due to the cell lines’ particular complexity and heterogeneity. The heatmap has rows representing genes from the BT-549 cell line vector set and the columns representing cells across the global population sorted by sample ID. Combining the positive and negative expression yields high specificity for the cell line compared to the global population. Positively expressed genes such as *MFAP2* highlight gene expression of published protein markers for epithelial-mesenchymal transition (EMT). Significant expression of genes like *VCAN*, coding for genes encoding for proteoglycan involved in adhesion and proliferation, as novel genes descriptive for this particular cell line [27]. These gene markers can be leveraged in identifying model systems for patient disease when overlaying patient datasets with our established cell line set. These markers highlight significant characteristics of the BT-549 cell line. BT-549 is repeatedly identified as a cell line of interest in breast cancer modeling for replicating disease proliferation and rapid progression [28–32]. Therefore, BT-549 provides an appropriate model for identifying diverse cell populations, including stem and metastatic potential.

### Evaluating stemness potential across BSCLA cell lines

**Fig. 3:**
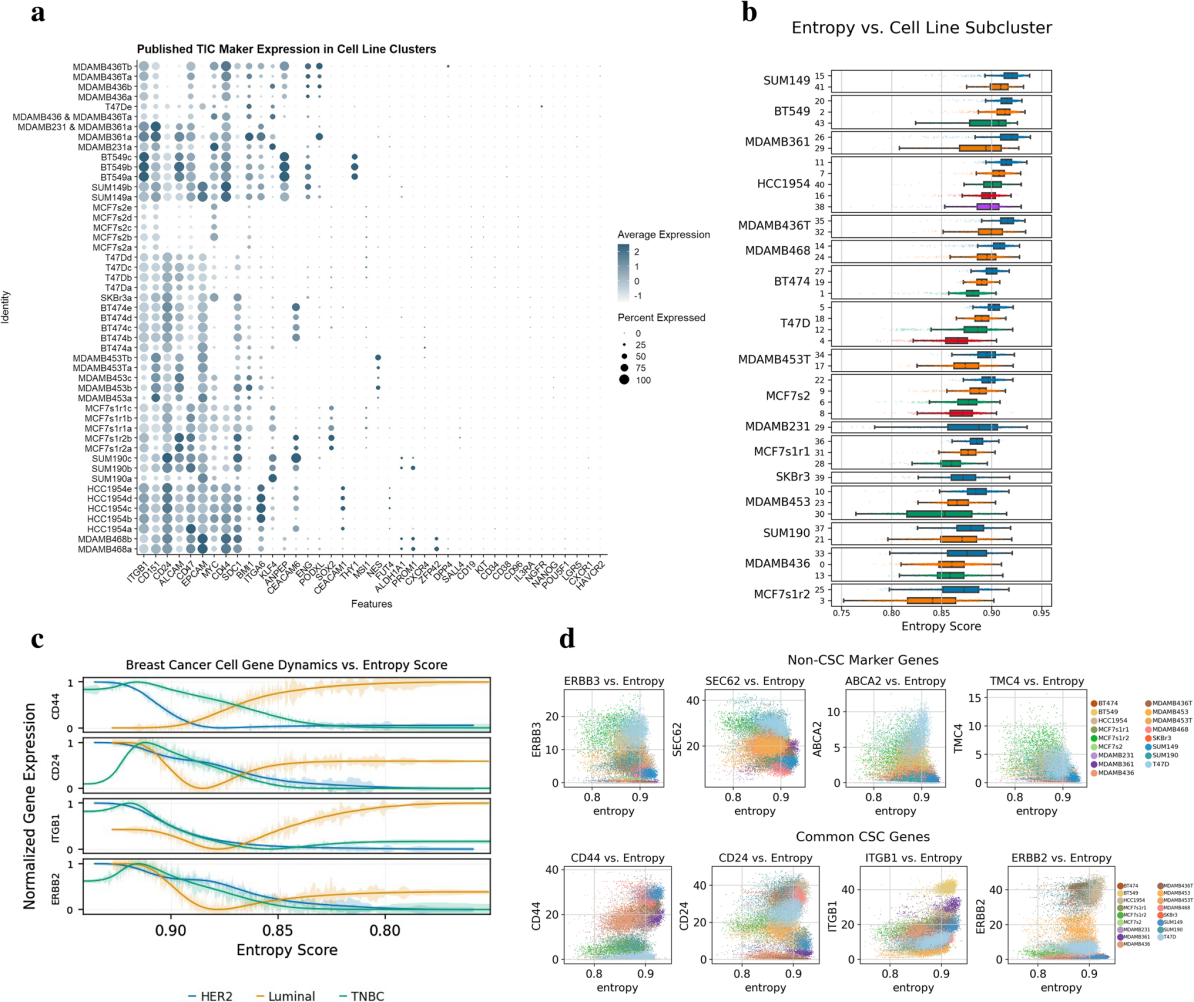
Investigating markers of Tumor Initiating Cells. **a** Dot plot visualizing expression of stemness-related genes identified through a literature review. **b** Box plot of cell lines and subpopulations sorted by mean entropy score. **c** Line plot comparing TIC marker expression by entropy score between major disease subtypes, where Luminal A and Luminal B samples were merged to a combined Luminal subset. **d** Scatter plots of key genes identified to have significant positive and negative correlation to entropy throughout the global dataset

One of the many use cases of BC cell lines, under normal or stimulated conditions, is the development and activity of stem/progenitor-like cells defined by specific gene regulatory networks important to cell line selection in oncogenesis and response to therapy [33, 34]. With our comprehensive BSCLA dataset, subpopulations were identified through CSC markers. Leveraging 40 established stemness markers across each cell line in the BSCLA, stem-like subpopulations were identified across the BSCLA cell line dataset. First, a comprehensive gene vector was generated using the known CSC markers (Supplementary Table 3) from published findings [35–37]. On the dot plot in Fig. 3a, subclusters were ranked by the sum total expression of the 40-marker vignette. Overall high concordance was observed in total CSC marker expression between intra-line subclusters, indicating a cell line of origin as the primary stemness effector. For example, in the MCF-7s2 population, all clusters yielded an average CSC expression with 3–3.5. However, heterogeneity is observed between intra-line subclusters, identifying these populations of interest for further investigation. For example, the “190c” cluster was observed to express a sum CSC value of 28.3, whereas the subclusters “190a” and “190b” have values of 10.8 and 20.3, respectively. This supports the theory of uncharacterized heterogeneity between intra-line subpopulations.

An entropy scoring approach was incorporated into gene expression profiles in estimating measures of differentiation. The Shannon Entropy measurement (see Section 2) quantifies levels of gene-to-gene interactions. With single-cell entropy scoring inversely correlating to degrees of differentiation, scores were extrapolated to identify stem-like populations and markers of interest. The final entropy scoring gradient plots are illustrated by the box and whisker plots shown in Fig. 3b. The cell lines are ranked by total entropy, and the subclusters are subsequently sorted by entropy reading within the cell line and subclusters. To illustrate the potential heterogeneity of entropy scores, the scores were overlaid on published CSC markers. These genes are published markers for CSC identification (*CD44*, *CD24*, *ITGB1*, and *ERBB2*). Expression was plotted versus entropy level while grouping with condensed disease subtypes (Luminal, HER2 + , and ERBB2), shown in Fig. 3c. The line plot for TNBC cell populations indicates a negative correlation between CSC markers and entropy score for all the markers plotted. Similarly, an inverse correlation is observed for the same genes in luminal disease. This scoring algorithm was leveraged to identify significant gene correlations with entropy values. *ACTN1* is identified as a marker with a significant positive Spearman correlation to entropy. Figure 3d highlights the expression of four high positive and four negative correlation markers in a scatter plot with entropy. These trends can be applied to derive features most associated with stemness across our population and highlight cell lines with significant variability in entropy between genes (i.e., BT-549 and SUM149). Through the current understanding of in vivo cancer cell differentiation, only one cluster within the population needs to reflect stemness capacity in gene expression for CSC identification. By sorting cell subpopulations by the overall presentation of our comprehensive CSC gene vector list on the subclusters resolution, cell lines were ranked by potential stemness, indicating each cell line’s potential for differentiation.

Both analyses yielded BT-549 as a cell line with strong CSC potential. This process causes the ranking order outlined in Supplementary Table 4 with BT-549 and MCF-7 as high and low stemness populations, respectively. The analysis was then reproduced through the entropy scoring algorithm to re-rank subclusters based on a complexity score. High concordance was observed in ranking between both our analysis methods, further verifying our stem-like subpopulations as lines of interest for further investigation.

### Analysis of merged single cell atlas data from breast cancer tissue states

**Fig. 4:**
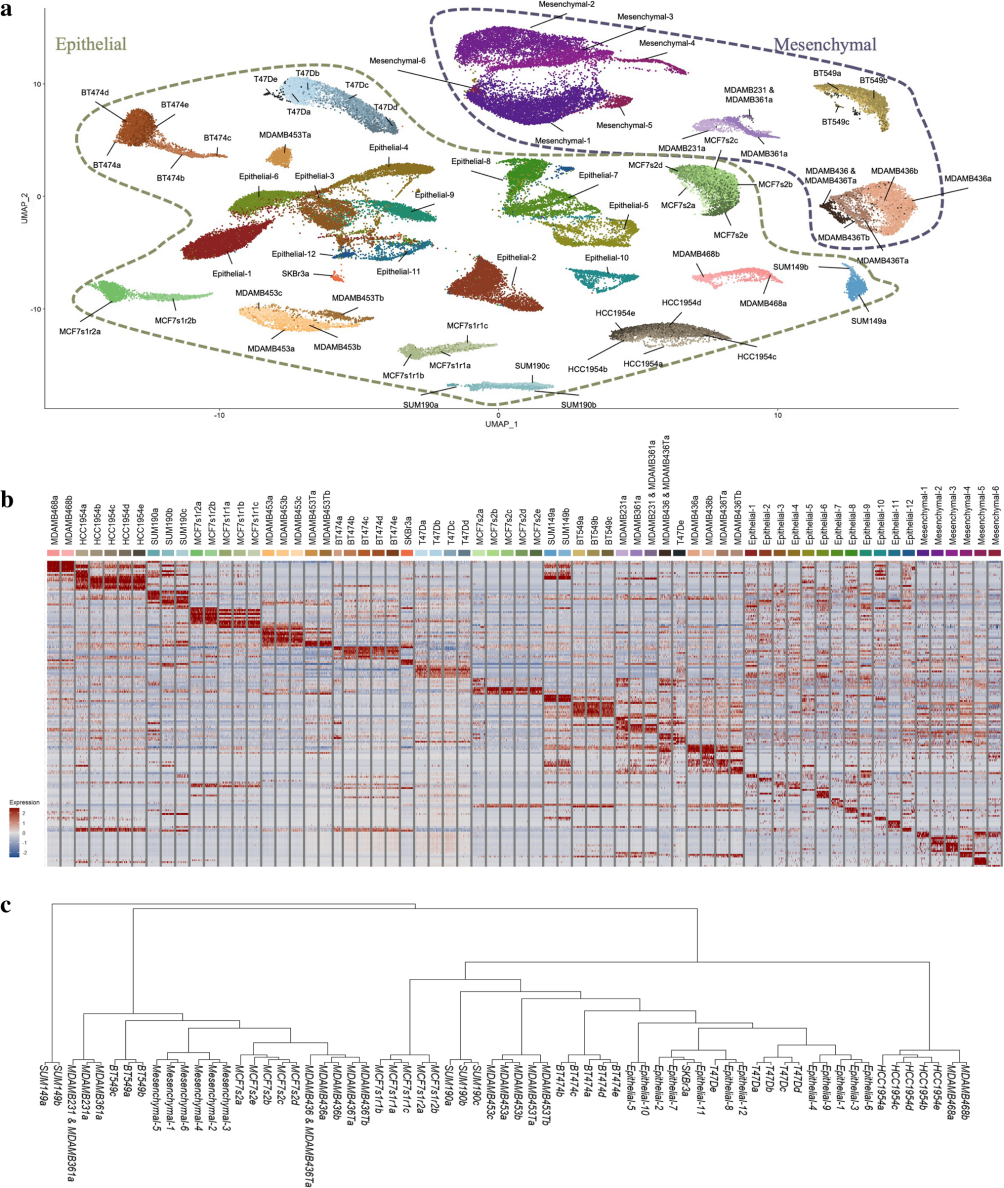
Merged Atlas Analysis from Publicly Available Tissue Dataset. **a** UMAP plot of merged datasets from cell lines and tissue sequencing. Tissue dataset was reclustered and annotated using published markers. Visualization indicates localization of Epithelial and Mesenchymal populations across cell line and tissue samples. **b** Heatmap plot of merged dataset with top genes identifying each subpopulation, highlighting gene level heterogeneity across population. **c** Phylogenetic tree outlining cluster differentiation. Localization of cell lines and tissue samples is observed with some cell line clusters embedded in tissue cluster branches, specifically T47De and SKBr3a populations

Wu et al. recently published scRNAseq, CITE-seq, and spatial data for primary tumor tissues representing various BC disease subtypes [38]. The study sequenced over 100,000 breast cells and provided the largest single-cell resolved atlas of BC tissue states. By integrating datasets between cancer biopsy cells and BSCLA cell lines, the value of selected cell lines for modeling patient tumors is demonstrated. After downloading the publicly available dataset from the Broad Institute Single Cell Portal, epithelial and mesenchymal cells were selected using cluster expression of canonical markers *EPCAM* and *PDGFRB*. These normal and cancerous cell types were the selection criteria for cell line development and, therefore the subset selected for model system comparison. Immune cells were filtered out for their limited relevance in cell line data comparison. Figure 4 summarizes the analysis conducted in the cell lines extended to the merged Seurat object containing cell line data with the primary tumor dataset. After the datasets were filtered for cancerous cell types, they were reclustered with a resolution of 0.6 to identify 12 epithelial and 6 mesenchymal clusters. A breakdown of clusters in the merged dataset and respective source sample is visualized by the stacked bar plot in Supplementary Fig. 1a**.** Similar to the phylogenetic tree visualizing cluster distances in cell lines, Supplementary Fig. 1a indicates distance relationships of each of the original primary tissue sample identifiers compared to the cell line samples. Figure 4a is a UMAP plot visualizing the merged dataset annotated for cluster identities with the precise organization between epithelial and mesenchymal cell types. Figure 4b is a heatmap highlighting differential genes expressed in each cluster from the merged dataset generated from top hits identified from the FindAllMarkers() function. Gene markers for the mesenchymal subtypes indicate less heterogeneity than the epithelial clusters, characterized by shared expression of multiple genes across the cell clusters, including *COL1A1*, *COL1A2*, and *TAGLN*. Top gene hits for each primary tumor dataset cluster are leveraged to perform functional network predictions for each subpopulation, illustrated by the cnet plots in Supplementary Fig. 2. Network analyses provide a population-based understanding of cell function. Clusters in epithelial cells and mesenchymal cells can parallel shared functionality within their ecosystem; for example, Epithelial-7 and Mesenchymal-2 highlight significant gene expression for pathways in negative regulation of cell proliferation. Similarly, Epithelial-5 and Mesenchymal-5 present and emphasize pathways involved in vesicle formation and regulation. Figure 4c is a phylogenetic tree that highlights distance relationships within clusters of the merged dataset. Clusters are organized by the sample of origin; however, we observe infiltration of cell line clusters in the tissue dataset clusters such as SKBr3a and T47De. These analysis techniques inform which model cell lines are relevant to specific cell functionality across BC subtypes.

To further investigate cell line versus patient tumor overlap and heterogeneity, HER2 expressing cell lines, BT-474 and HCC1954, were merged with HER2 expression patient sample CID3921. The HER2 merged dataset was merged using Harmony, a pipeline to scSeq data merging that encourages mixed data representation in clusters from samples [22]. The reclustered population breakdown yielded 5 unique populations, visualized by the bar plot in Supplementary Fig. 3a. Clusters are primarily separated by sample origin ID despite data integration and normalization, with cluster HER2-0 sourced primarily from HCC1954 (99.4%), HER2-1/HER2-2 primarily from BT-474 (97.4% and 92.4%, respectively), and HER2-3/HER2-4 predominantly from CID3921 (96.3% and 100%, respectively). Supplementary Fig. 3b and c provides further resolution on sample source distribution against a UMAP plot paralleled with cluster identification. Cell lines BT-474 and HCC1954 generate two distinct partitions with patient data from CID3921 present as its own partitions and contributing to the other two cell line functional partitions. Genes present throughout this HER2 subsetted population were parsed for significant and consistent expression across the dataset, generating 124 significantly conserved genes across all 5 clusters. Some of the conserved genes, *SERBP1*, *S100A10*, *ARF1*, and *PRDX1*, are plotted by the RidgePlot in Supplementary Fig. 3d. Gene expression of *S100A10* has been linked with metastasis and stemness, while *ARF1* is indicated as a regulator of cell proliferation [39, 40]. The expression of these proliferative gene signatures in HER2 + samples supports previous findings and the inherent invasive biology within HER2 disease. The FindAllMarkers() function run against sample identifiers and cluster identifiers yielded gene vectors specific to sample sources and clusters. Top hits from each vector, sorted by Avg_logFC are visualized by the heatmaps in Supplementary Fig. 3e and f. Differential genes expressed by the patient tumor data include *GNB2L1*, which serves as a prognostic marker, inducer of proliferation, and potential drug target in breast cancer [41]. Across the same analysis, we identify DEGs specific to the patient sample, including *GPX1, GSTP1*, and *CALML5*. Similarly, DGEA reveals gene signatures specific to each cell line not expressed by the other line or patient tumor data, including *TFF3, MDK,* and *KRT19* isolated to BT474 and *S100A9, LCN2,* and *HLA-B* specific to HCC1954. While clusters are primarily sourced from an individual sample, representative cells from the patient data are present in partitions from both cell lines. 437 of 603 cells from CID3921 are populating the cell line functional partitions, indicating functional resemblance to patient samples with representative cell lines.

### Unsupervised cell type prediction through custom unsupervised cell annotation pipeline

**Fig. 5:**
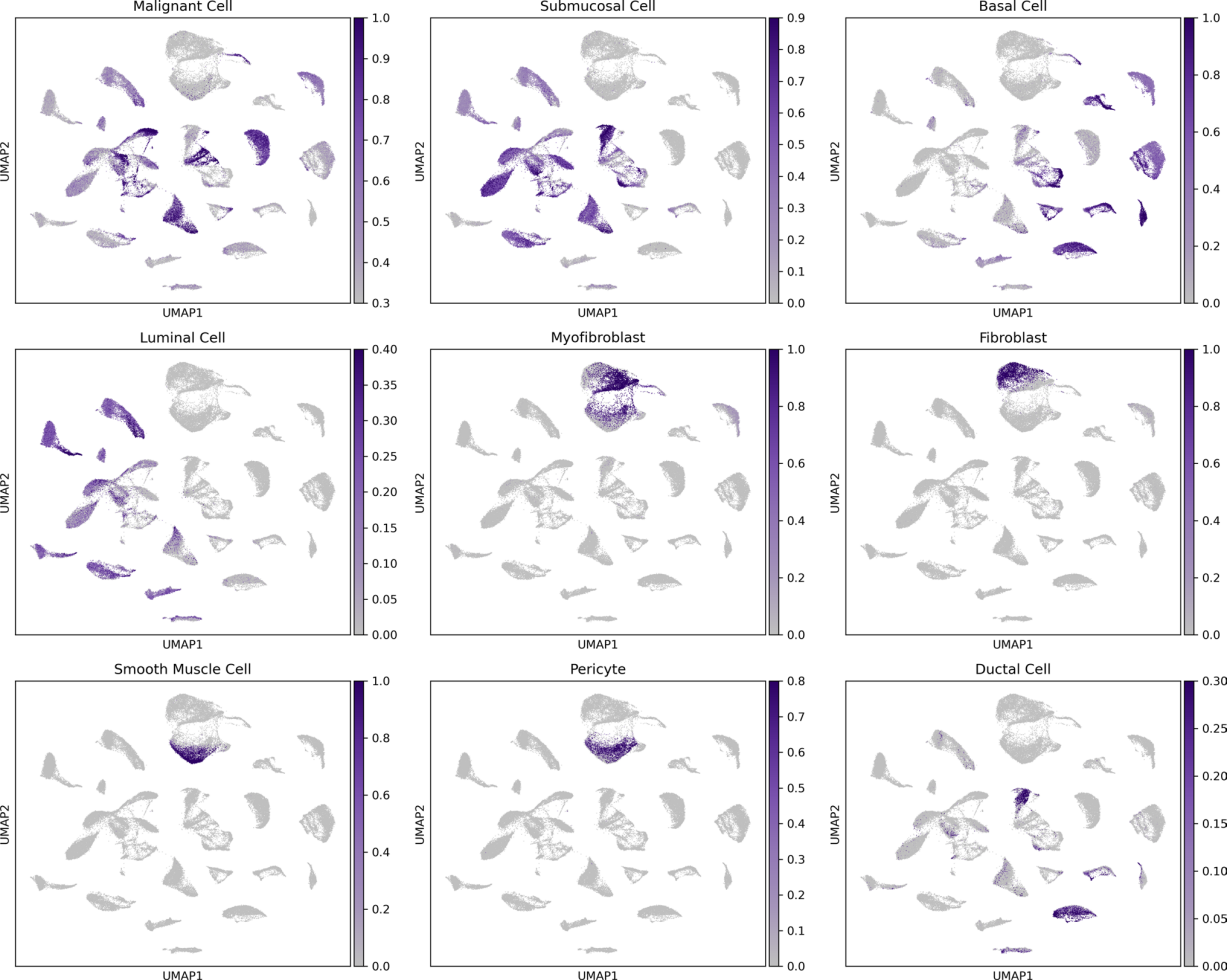
Unsupervised classification of merged dataset. UMAP plots of top identified cell types across the merged dataset. Basal and ductal cells are representative of the functional cells illustrated previously in Fig. 1, where expression of these functional cell types is observed in both primary tumor samples and cell lines

Cells were further processed through an unbiased annotation platform to understand the functionality of cell clusters from the integrated dataset. The platform integrates single-cell annotations from over 28 million cells across tissue types and cell lines to predict cell type. The top hits generated across the dataset are illustrated with the UMAP plots in Fig. 5. Cell type-specific gene annotations for populations of cells typical of breast tumor tissue, including malignant cells, basal cells, luminal cells, myofibroblasts, fibroblasts, smooth muscle cells, pericytes, and ductal cells, were provided. Some cells identify as submucosal cells, typically present in the airway. These cell phenotypes resemble cells necessary near surface epithelium for mucin secretion and antimicrobial host defense within the breast ductal network [42, 43]. Cell type predictions with the highest observed significance included malignant cells, basal cells, myofibroblast, fibroblast, and smooth muscle cells. Both BC tissue and cell line data overlap in predicted cell type for various annotations, including malignant, submucosal, basal, luminal, and ductal cells.

### High-resolution subtype classification of cell clusters using gene expression data

**Fig. 6:**
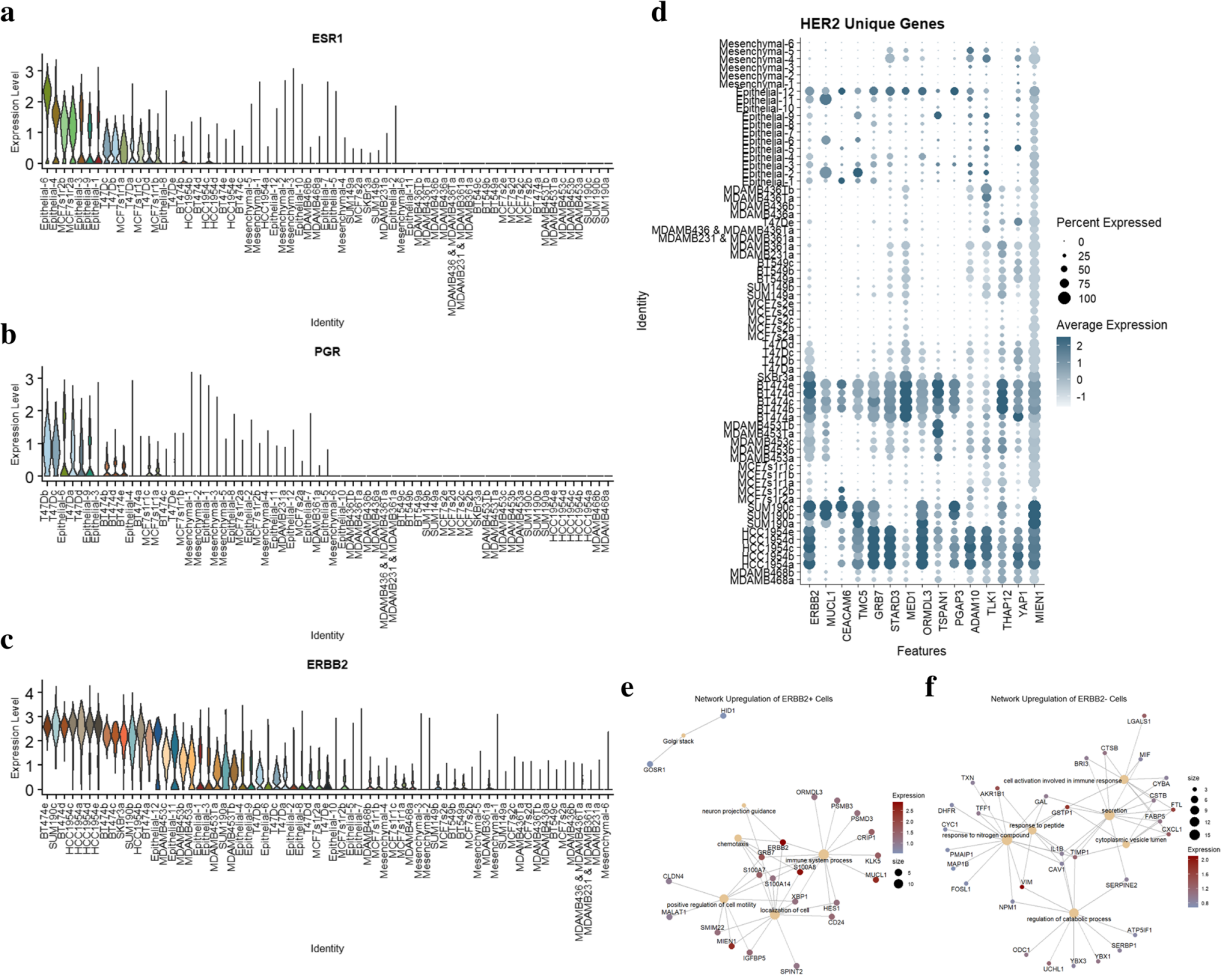
Higher resolution subtyping of breast cancer cell lines and HER2 characterization. **a** Violin plot outlining expression of hormone receptor gene ESR1, with high expression observed in epithelial primary tumor cell clusters. **b** Violin plots outlining expression of hormone receptor gene PGR. **c** Violin plot outlining expression of receptor genes ERBB2. **d** DotPlot outlining expression of genes identified as differentially expressed by HER2 + cell samples. **e** Network analysis of genes differentially expressed by global ERBB2 + cells. **f** Network analysis of genes differentially expressed by global ERBB2- cells

Breast cancer disease subtypes are categorized by ER, PR, and HER2 expression levels. There is increasing indication that through higher resolution data and novel marker discovery, this current bulk method of subtyping can be improved upon [44]. Like disease subtyping, our model systems are characterized by the same expression markers in breast cancer and are also vulnerable to potentially outdated or inconsistent classifications. There is published variability in subtype identity across individual cell lines [45, 46]. This is due to variability in processing and analysis, including lab level discrepancies in protocols for identification, including gene versus protein, expression versus overexpression, and culture conditions contributing to the population differences. With higher resolution, scRNAseq data, comparing gene expression differences across cell identities in an individual cell line and tissue dataset developed more informed subtyping. This serves as a precursor to further validation on the protein level. The threshold of expression as the quantitative split observed in bimodality of expression between detected expression and overexpression is identified at 0.25. Figure 6a, b, and c are a series of violin plots visualizing the expression of the *ESR1*, *PGR*, and *ERBB2* genes, respectively. This analysis creates a higher resolution subtyping of cell lines and cancer tissue datasets by gene expression. Higher-resolution disease classification is concordant with published annotation for many cell lines. For cell lines MDA-MB-361, MDA-MB-453, and SUM190, the expression of *ESR1*, *PGR*, and *ERBB2* showcase heterogeneity in expression either between clusters or with a published characterization of these samples for their disease subtype. The MDA-MB-361 cell line is typically categorized as HER2 + or Luminal in previous studies [9]. However, no significant expression of the critical gene markers defining Luminal and HER2 + disease subtypes is expressed. Further single-cell analysis with linked protein analysis such as a CITE-seq can resolve these findings for this cell line. When comparing the expression of *ERBB2* between subpopulations in a cell line, multiple lines indicate heterogeneity in expression between clusters. In MDA-MB-453 and SUM190, there are subclusters for both cell lines with *ERBB2* overexpression and clusters with average/low expression. There is one cluster in MDA-MB-453 (cluster c) and SUM190 (cluster c) with an observed lack of expression of ERBB2, with 78% and 64% of the cells in those clusters expressing this gene below a significance threshold, respectively. The discrepancy in the expression between these populations can be the confounding source between the published classifications for these cell lines. Most clusters from the breast cancer biopsy cells align with a specific disease subtype pattern. Epithelial-6 and Epithelial-9 are classified as luminal attributed to high hormone receptor gene expression but lack *ERBB2* overexpression. The Epithelial-1, Epithelial-2, Epithelial-3, Epithelial-4, Epithelial-8, Epithelial-9, Epithelial-11, Epithelial-12 tissue clusters align with the HER2 + breast cancer subtype. 38.8% and 63.0% of cells across the entire merged dataset and the confirmed HER2 + clusters, respectively, express *ERBB2* at a scaled value above 0.25. Clusters Epithelial-5, Epithelial-7, Epithelial-8, Epithelial-10, Mesenchymal-1, Mesenchymal-2, Mesenchymal-3, Mesenchymal-4, Mesenchymal-5, and Mesenchymal-6 didn’t detect significant expression of the diagnostic genes and therefore are classified as TNBC. High concordance is observed between epithelial TNBC clusters and a CellNet basal prediction, further validating the role of trained unsupervised cellular annotations in scRNA datasets. Supplementary Table 5 annotates the percent of cells in each cluster expressing each diagnostic marker and respective average expression. Due to the resolution of scRNAseq, this assay develops a higher resolution characterization of breast cancer cell lines within the current subtyping system (Table 2). It resolves the source of heterogeneity for some of the confounding cell lines on the gene level.

**Table 2:** Characterization vectors for each processed BC cell line

Cell Line	Previously Published subtype	Cell subtype	Tumor	Location	ER	PR	HER2	BRCA1	Heterogeneity	Stemness	High-resolution subtyping	Marker Identification
SKBr3	HER2 +	Epithelial	AC	Metastasis	-	-	+	-	2	7	HER2 +	CTAG2
MDA-MB-361	HER2 + / Luminal B	Epithelial	AC	Metastasis	+	±	+	-	4	2	TNBC	SRGN
BT-474	HER2 + / Luminal B	Epithelial	Invasive DC	Primary	+	+	+	-	3	9	HER2 +	FAM3B
MDA-MB-453	HER2 + / TNBC / Unclassified	Epithelial	AC	Metastasis	-	-	+	-	3	10	HER2 + & TNBC	ANKRD30A
MCF-7	Luminal A	Epithelial	Invasive DC	Metastasis	+	+	-	-	4	4	Luminal	TFF1
T47D	Luminal A	Epithelial	Invasive DC	Metastasis	+	+	-	-	5	6	Luminal	SERPINA6
MDA-MB-468	TNBC	Epithelial	AC	Metastasis	-	-	-	-	3	12	TNBC	SLC1A6
SUM190	TNBC / HER2 +	Epithelial	Inflammatory DC	Primary	-	-	+	-	3	11	HER2 + & TNBC	ALDH3A1
HCC1954	TNBC / HER2 +	Epithelial	DC	Primary	-	-	+	-	3	13	HER2 +	FAM9C
SUM149	TNBC	Epithelial / Mesenchymal	Inflammatory DC	Primary	-	-	-	+	2	8	TNBC	WFDC2
BT-549	TNBC / Claudin-Low	Mesenchymal	Invasive DC	Primary	-	-	-	-	5	5	TNBC	COL1A2
MDA-MB-436	TNBC / Unclassified	Mesenchymal	AC	Metastasis	-	-	-	+	4	3	TNBC	MAGEA4
MDA-MB-231	TNBC / Unclassified / Claudin-Low	Epithelial / Mesenchymal	AC	Metastasis	-	-	-	-	2	1	TNBC	SRGN

This table is a summary of current publicly available understandings of each cell line as well as novel identifiers and characterization vectors from the analyses discussed here. Heterogeneity is a ranking system based on cluster counts generated from a standard resolution parameter. Stemness is the ranking score based on custom entropy scoring predictions. Higher-resolution subtyping clarifies some of the ambiguating in cell line disease subtyping. Marker identification is a significant and isolated representative gene in each cell line compared to the remaining global dataset

### Evaluating single cell protein and gene expression of ERBB2 in MDA-MB-453

To further evaluate the association and heterogeneity of gene expression and protein expression within breast cancer cell lines, the MDA-MB-453 cell line was processed for high-throughput microfluidic microscopy. This is one of the cell lines that, in publications, and our higher resolution gene analysis, indicated heterogeneity in the expression of ERBB2 [11]. The expression of ERBB2 on MDA-MB-453 cells was measured using both traditional fluorescent microscopy, and a microfluidic chip was used that allows isolated single-cell imaging and functional experimentation. Within the microfluidic system, a total of 1219 nanopens were population with MDA-MB-453 cells. Of those pens, 1103 contained an individual cell, and 116 contained multiple cells. The data from this imaging system is processed through a MATLAB script where 1147 cell locations are identified using a Hough Transform circle detection algorithm. The 72 cells penned that were not found by the algorithm could be due to a multitude of image factors, including cell brightness not passing the traditional threshold of live cells, masking due to chip background interference, and location filtering due to cell import and localization near the top of a nanopen. Image analysis of the MDA-MB-453 cell lines indicates heterogeneous surface marker expression across the population. ERBB2 expression is quantified in 1147 detected cells. Through this analysis, 513 (44.7%) of 1147 cells had a significantly positive expression of ERBB2. The violin plot in Supplementary Fig. 4a quantitates the normalized FITC brightness measurement across all cells detected on the microfluidic chip imaging platform. In the microscopy imaging assay using, 87 (53%) of the 164 MDA-MB-453 cells imaged indicated positive expression based on a scaled threshold of 0.5. Supplementary Fig. 4b is a violin plot of ERBB2 expression of 164 MDA-MB-453 cells imaged through traditional microscopy. We observe this variability of surface marker expression concordant with heterogeneity in single-cell gene expression of ERBB2. 2050 (88.0%) of 2330 MDA-MB-453 cells were identified as positive for ERBB2 gene expression from the scRNASeq data (Supplementary Fig. 4c). Supplementary Fig. 4d is a series of sample microscopy images captured overlaying expression with brightfield cell images. Supplementary Fig. 4e is a series of images sampling 40 nanopens with cells detected as ERBB2 positive and ERBB2 negative on the microfluidic system. Normalized expression scores are listed below each cell detection.

### *HER2* + *BC cell gene concordance provides insight toward disease progression*

A high-powered gene analysis between subtypes is generated by leveraging the higher resolution subtyping of breast cancer model lines. HER2 + is a breast cancer subtype defined by overexpression of the HER2 protein or *ERBB2* gene. By identifying cell lines and breast cancers, tissue cell clusters that classify as HER2 + through consistent and significant gene expression in their population (pct > 0.5 with expression > 0.5), assisted gene sets are developed for this relevant disease subtype model system. To do so, the filtered Seurat objects representative of BT474, HCC1954, SKBr3, SUM190, MDA-MB-453, Epithelial-1, Epithelial-2, Epithelial-3, Epithelial-4, Epithelial-8, Epithelial-9, Epithelial-11, and Epithelial-12 are merged to generate a HER2 + population-level object generated from our re-annotated grouping. A similar analysis is done to create a non-HER2 + Seurat object composed of cell datasets from the MCF-7 samples, T47D, MDA-MB-468, BT-549, SUM149, MDA-MB-36, MDA-MB-436, MDA-MB-231, and Epithelial-5, Epithelial-6, Epithelial-7, Epithelial-8, Epithelial-9, Epithelial-10, Mesenchymal-1, Mesenchymal-2, Mesenchymal-3, Mesenchymal-4, Mesenchymal-5, and Mesenchymal-6. These populations’ datasets are normalized and scaled to optimize data analysis that otherwise can get distorted with larger sample sets with varying quality metrics. The HER2 + object and non-HER2 + objects were independently reclustered and annotated. The FindAllMarkers() function on Seurat V3.0 (see Section 2) gives a comprehensive list of genes across the subclusters defining the HER2 + cell line population, and another gene set for the non-HER2 + cell line set is generated. Using inverse intercept filtering identifies genes significantly and frequently expressed in the HER2 + population (pct. > 0.4). This gene vector is then intersected with the vector of genes significantly expressed (pct. > 0.2) in the non-HER2 + population. The gene set defining the HER2 + population are anti-joined to the new intersected gene sets. Each gene set is filtered out to remove ribosomal and mitochondrial influence. This yields a vector of 323 more highly expressed genes in HER2 + cell clusters than non-HER2 + clusters, with some markers lacking detection in the non-HER2 + cells. While lack of detection can also be due to the failure of the assay to capture representative RNA, it remains an indicator of a low-expressed molecule in the non-HER2 + cell lines and clusters. Figure 6d is a DotPlot highlighting the heterogeneity in frequency and intensity of expression for 13 genes in the gene vector delineating positive expression in HER2 + and reduced expression in non-HER2. The genes highlighted by this analysis corroborate published markers for breast cancer, such as *MUCL1* and *CEACAM6*. One finding with high concordance is coordinated amplification of HER2-neighboring genes on the same amplicon, including *GRB7*, *PGAP3*, and *MIEN1* [47].

A Gene Set Enrichment Analysis (GSEA) is run across the HER2 + BC cell lines using the ClusterProfiler R package. The input to this pipeline is a data frame of driving genes identified through the FindMarkers() function on Seurat V3.0 (see Section 2) comparing ERBB2 positive cells against ERBB2 negative cell identifiers. Figure 6e is a cnet network plot identifying gene pathways upregulated in the global ERBB2 positive dataset compared to ERBB2 negative. A significant (GeneRatio > 0.5) activation of critical pathways contributing to HER2 + cancer disease progression, including regulation of cell motility, chemotaxis, and neuron projection guidance, is observed in ERBB2 positive cells. Figure 6f is a cnet network plot identifying gene pathways upregulated in the global ERBB2 negative dataset. This highlights a significant (GeneRatio > 0.5) activation of cytoplasmic vesicle lumen, cell activation involved in immune response, response to nitrogen compound, and regulation of catabolic processes. As indicated, there is an overwhelming pathway activation involving *MUCL1*, *S100A8*, *S100AS7*, *S100A14,* and *MIEN1* in HER2 + cells. These findings support current developments in HER2 + progression where Y I Bao et al. identified that *S100A8* induces downregulation of estrogen receptor (*ESR1*) and is thereby a mechanism for poor prognosis in HER2 + cancers [48]. Li et al. investigated *MUCL1* as an influencing factor in cell migration and invasion within breast cancer cells. Their functional work showed that knockdown of the gene in the MCF-7 and MDA-MB-231 cell lines resulted in decreased migration and invasion, whereas overexpression had the opposite effect [49]. Similarly, Sneh et al. showed S100A7 expression enhances EGF-induced actin remodeling and increases metastasis compared to control in MDA-MB-231 [50]. These published functional experiments support the correlative findings of HER2-expressing cells. There is noted activation of novel pathway markers such as *PSMB3*, a component of the 20S proteasome complex responsible for protein homeostasis involved in all the top 5 activated pathways within the HER2 + population, including positive regulation of defense response.

### Published marker expression patterns in merged atlas dataset

Wu et al. developed four gene vectors characterized through pairwise integration between PAM50 subtypes and a 2,000-gene intrinsic list from TCGA. These gene vectors established standardized molecular subtypes of primary breast cancer. Each gene vector is overlaid for expression across the merged dataset with Supplementary Fig. 5a, 5b, 5c, and 5d, indicating expression of specific genes in each gene vector for HER2, Luminal B, Luminal A, and TNBC, respectively. In Supplementary Fig. 5a, high concordance is observed with HER2 correlated genes with the expression of HER2 classified populations, including BT-474 and HCC1954 cell lines. Heterogeneity in disease subtyping across cell lines is supported by variable expression of specific genes from the correlation network on cell lines such as MDA-MB-453 and SUM190. For example, SUM190a is the cluster with the lowest expression of gene ERBB2 and consistently has lower expression of genes in the correlation network for HER2 than SUM190b and SUM190c. This pattern is illustrated by genes *ID3*, *MED24*, *GRB7*, and *PGAP3*. Similarly, the gene vectors for Luminal A and Luminal B are supported by positive expression of many markers in our cell line populations, including some MCF7 clusters with expression of *STARD10*, *C6orf48*, and *TFF1* from Luminal B and *HSPB1*, *KRT8*, and *PPDPF* from Luminal A, visualized in Supplementary Fig. 5b and 5c. MCF72 clusters display positive expression of TNBC vector genes rather than the expected Luminal gene sets. These gene signatures parallel the expression of tissue sample clusters Epithelial-10, Epithelial-11, and Epithelial-12. Supplementary Fig. 5d highlights gene expression of TNBC gene vectors with the highest concordance in cell line data with BT-549 clusters that express *UCH1*, *CDKN2A*, and *CAV1*.

### Functional analyses of BT-549 and MDA-MB-436 cell line subpopulations

Cell differentiation is identified by component molecules that define cellular functionality. Through scRNAseq, cellular compartmentalized expression profiles were derived that can be used to predict perturbations in pathways of potential interest for protein level comparisons. Through comparing these predictions with known cell types and states, cellular function predictions are made across majority and minority subpopulations. As discussed above, Seurat objects are independently normalized and scaled within individual cell line samples. Individual cell line analysis provides a deeper understanding of observed and novel heterogeneous population responses. While each line was investigated for functional heterogeneity, the BT-549 and MDA-MB-436 lines represent populations with high and low stemness potential, respectively, determined by sorting lines by sum expression of 40 CSC markers. The functional differences observed within these lines provide a necessary understanding of model systems and their implications as first-of-line investigative tools for therapies and toxicity. Both cell lines were independently reclustered to leverage the higher resolution dataset and account for local difference levels versus prior global resolution.

MDA-MB-436 is a TNBC cell line composed of mesenchymal type cells. When locally reclustered, the MDA-MB-436 line has four subclusters shown in Supplementary Fig. 6a. Gene vectors defining each subpopulation, outlined in the heatmap Supplementary Fig. 6b, drive functional heterogeneity predictions. These genes were sorted by Avg-logFC from a comprehensive list generated for markers throughout the MDA-MB-436 cell line population. Our preliminary analysis with these gene sets provided interesting gene expression patterns for many populations. Subpopulation 0 has a high expression of *SAA1* and *IL1A*, both immune response signals generated in response to tissue/cell injury. Subpopulation 1 is defined by the unique expression of *IFIT1*, *IFIT2*, and *IFIT3*, which are all part of a tertiary complex generated to resist viral pathogenesis in host cells. Subpopulation 2 has significant expression of *RAD23A* and *NDUFB7,* which are both involved in the ubiquitination process and other genes responsible for nucleotide excision repair. Subpopulation 3 in MDA-MB-436 has a high expression of *DDIT3* and *TXNIP*, which are both markers of a cellular stress response. ZFAS1, another feature of this subpopulation, is associated with cancer progression and metastasis. While most of these gene sets can predict cell states and functions, they still require further validation to verify these cell properties. Each subpopulation in the MDA-MB-436 sample expresses varying forms of a cell stress response. Supplementary Fig. 6c is a RidgePlot quantifying the expression of conserved markers with the cell line, filtering out ribosomal genes as they are typically ubiquitously expressed in healthy cells. These genes have varying supporting functions expected from a cancer cell population, including, for example, supporting cell proliferation (*NPM1*) and telomere maintenance (*NHP2*). This gene-level heterogeneity inputs to a more extensive pathway analysis using the R package ClusterProfiler. After generating gene vectors defining divergence in intra-line subpopulations, GSEA was run. This analysis identifies overlapping gene sets which provide functional modules based on top differentially expressed genes between populations. Supplementary Fig. 6d is a cnet plot highlighting pathway nodules significantly upregulated in the MDA-MB-436d subpopulation. This analysis indicates four pathway hits: intracellular organelle lumen transcription, cell surface receptor signaling pathway, DNA-templated transcription, and nucleolus. While many of these pathways are already of interest in cancer development and progression, this enrichment is localized to a cluster within the MDA-MB-436 line.

BT-549 is a TNBC epithelial cell line from a ductal carcinoma. The cell line dataset was locally reclustered to yield six unique subpopulations within the line. BT-549a constitutes 2527 cells from the 2748 (91.9%) total cells sequenced and passing QC filters of the cell IDs. This subpopulation would significantly dominate the bulk analysis of the cell line. This is supported by gene expression of critical genes in breast cancer pathology, such as *AR,* the androgen receptor gene. BT-549 is identified as a cell line with high expression of the *AR* gene: however, expression (> 0) is detected in 1,753 of 2,748 (63.8%) cells [51]. Repeating the analysis performed for MDA-MB-436, Supplementary Fig. 6e, 6f, 6 g, and 6 h represent BT-549 to showcase a UMAP, heatmap of a subset of cells representing each subpopulation with gene vectors for each cluster, a RidgePlot for the conserved genes across the cell line and a cnet network plot generated from top differential genes from BT-549e. The heatmap provides unique functional predictions for many of the subpopulations within our cell line: *SRGN* expression in subpopulation 1 indicates CSC properties, *POLR2L* and *ROMO1* expression in subpopulation 2 highlights a transcriptionally active cell state in proliferation, *ITGA10* expression in subpopulation 3 highlights a potentially metastatic subpopulation further supported by *DYNC1H1* expression, *PPP1R15A* expression in subpopulation 4 showcases a tumorigenic cell population recovering from stressful growth, and *CA2* expression in subpopulation 5 indicates cell population with poor prognosis with high energy consumption characterized by *CHCHD10* expression. The RidgePlot highlights conserved genes across these heterogeneous cell populations, filtering our ribosomal genes. These genes indicate cancerous populations by significant expression of *SERBP1* and *LDHB* important for mRNA stability and alternative energy consumption, respectively. Supplementary Fig. 6 h is a cnet plot highlighting pathway nodules significantly upregulated in the BT-549e subpopulation. This analysis indicates three overwhelming pathways: blood vessel development, positive regulation of response to stimulus, and negative regulation of phosphate metabolic processes.

When running a global FindClusters() function from the Seurat analysis pipeline (see Section 2) across our entire BSCLA dataset, a higher resolution parameter (3.0) is leveraged to account for the increased cell population. However, a lower resolution parameter (0.2–1.2) is used when processing individual cell line samples. By doing so, higher cluster counts for both cell lines are identified in local clustering compared to global. MDA-MB-436 and BT-549 generate 4 and 6 local clusters, respectively, compared to the 2 and 3 clusters from the global analysis. By selecting a low-resolution parameter (0.2) when sub-clustering locally, the analysis drives the selection of true heterogeneous populations despite also outputting an increased cluster count. Each line is independently analyzed to identify functional heterogeneity within these cell lines. This is better formulated through a lower resolution clustering parameter yielding larger subpopulations with more divergent gene sets. Through local cell line analyses, intra-line and inter-line variability is revealed. For example, cell lines like MDA-MB-436 that, despite representation from large subpopulations, the functional heterogeneity between them is minimal. In contrast, lines like BT-549 have significant functionally heterogeneous cell populations, many of which seem to drive disease progression and are easily obscured by bulk analysis due to overwhelming percentages of a particular cluster. Data for each of the remaining cell lines are published for open analysis as a tool for researchers to select lines best modeling their investigative needs. To make cell line selection better informed for the field, cell lines are organized by scoring for heterogeneity and stemness.

### Characterizing population variability between MCF-7 lab cultures

The MCF-7 cell line is of particular interest as it is one of the most frequently investigated lines with the highest source of data generation for patient care than any other breast cancer line [52]. MCF-7 is a luminal cell line commonly used for estrogen receptor investigation. There is agreed upon but uncharacterized clonal variation that is believed to maintain presence throughout culturing due to intercellular signaling [53]. There are also published observations of stem cells capable of populating the various cell identities [54]. To resolve some of the outstanding questions, three total samples of MCF-7 cell culture from two different lab cultures were processed, where samples from one lab were isolated at multiple passages (P2 and P6). A merged Seurat object is generated from the pre-filtered sample sets, then normalized and scaled to level sequencing depth and reduce technical variability. Cell cycle markers were regressed to reduce cell state heterogeneity confounding functional differences between populations. A UMAP plot is generated for the merged cell dataset (Supplementary Fig. 6i) and clustered at a medium resolution (0.6) to balance true populational differences and reduce noise effects. The clustered UMAP is shown in Supplementary Fig. 6j. Supplementary Fig. 6k is a phylogenetic tree comparing populational distances where the visualization highlights most clusters branch near their sample origin. Clusters branch with their culture of origin, except for cell subcluster MCF-7s1r1d – representing the MCF-7 cell line from sample source 1 from run 1 with cluster ID “d.” Additionally, in the UMAP Supplementary Fig. 6j on the top right, the small subclusters MCF-7s1r1d doesn’t plot near its sample source nor another lab culture. A gene set is generated for each subpopulation to understand functional differences between subclusters and the potential heterogeneity between samples. Based on differential expression of all markers, the genes were then sorted by difference value with the highest difference corresponding to genes with the most difference in the percentage of cells expressing it in a population compared to the remaining subpopulations. The top genes, sorted by Avg_logFC, are visualized in the heatmap Supplementary Fig. 6 l. These genes highlight inter-sample and intra-sample discordance. While many epithelial and mesenchymal cell markers are expressed by cells within the MCF-7 dataset, significant expression of mesenchymal markers *VIM* and *CDH2* is limited to the MCF-7s2 sample and the MCF-7s1r1d subcluster as well as isolated expression of epithelial marker *KRT19* on samples MCF-7s1r1 and MCF-7s1r2. This data supports the hypothesis that sample 2 has transitioned from epithelial to mesenchymal, whereas only a small subpopulation from sample1-run1 has transitioned. This supports the hypothesis that discrepancy is observed due to lab variability in culture methods and conditions, and may be attributed to cell line contamination. On a bulk level, this inter-sample heterogeneity would still be observable; however, the rare cell population representing MCF-7s1r1d would have been confounded by the other subpopulations. This further highlights the need for high-resolution data generation on commonly employed model systems. Functional differences between the identified populations can be resolved through evaluating canonical gene markers. For example, *PSCA* expression in MCF-7s1r2 is interesting as it is a stem cell antigen marker in prostate cancer. In the same sample, high gene expression of *BCAS1*, a gene marker that has not been observed in previous studies on MCF-7, is observed. Significant gene markers provide biological interpretations such as *AREG* in the MCF-7s1r1c subpopulation, a gene involved in estrogen action and ductal development. In cell line sample MCF-7s2, there is a significant expression of *GSTP1,* which has been shown to be expressed in drug-resistant MCF-7 cells [55]. Through analyzing these blunt gene patterns, a pattern of functional heterogeneity between samples of the MCF-7 cell lines is revealed.

### Prediction of potential therapeutic interventions leveraging targetable surfaceome

**Fig. 7:**
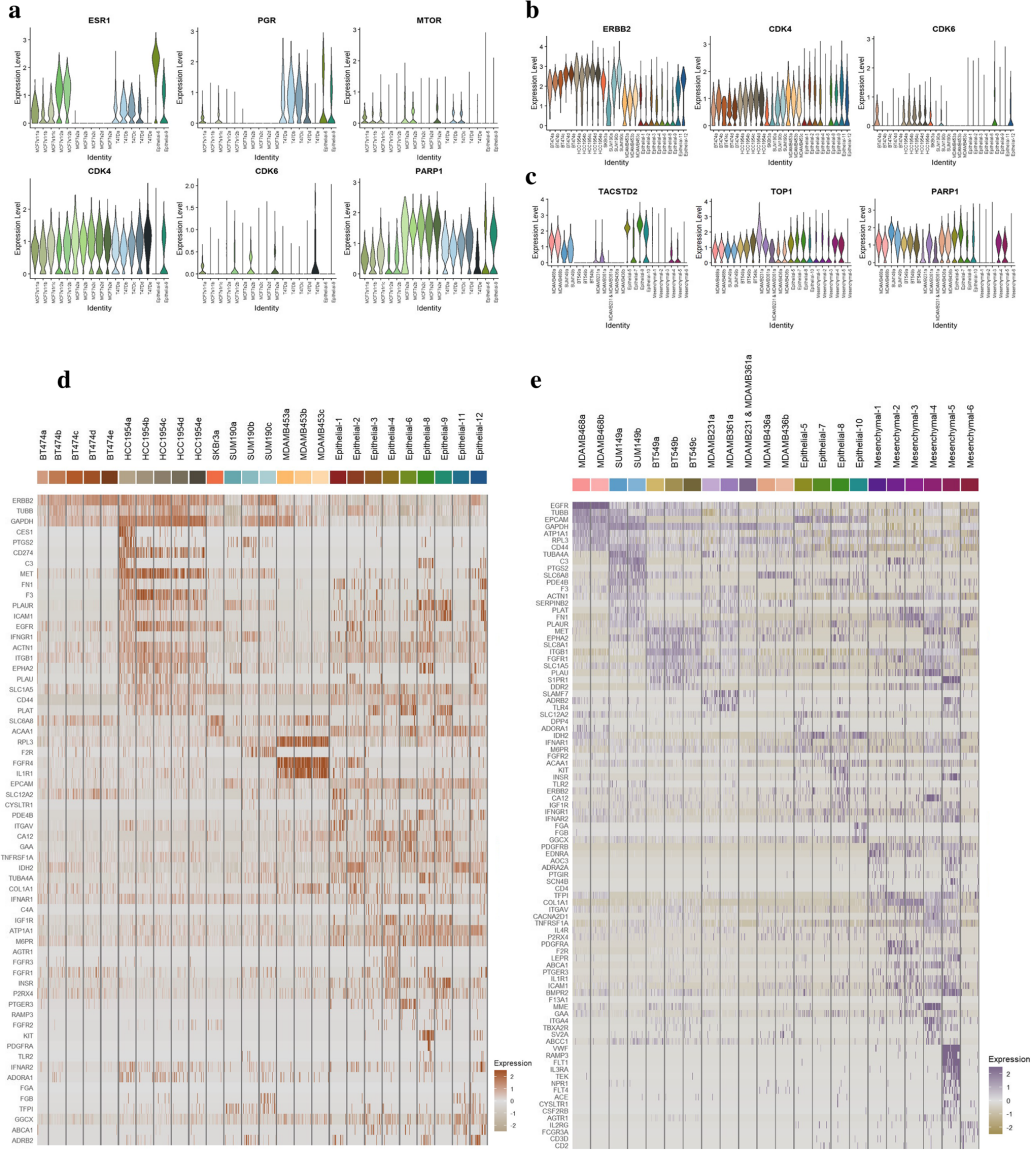
Predicting therapeutic response and novel therapeutic target prediction from scRNASeq gene expression. **a** Violin plots outlining drug targets and pathways for Luminal disease. **b** Violin plot outlining drug targets and pathways for HER2 + disease. **c** Violin plot outlining drug targets and pathways for TNBC disease. **d** Heatmap for HER2 + specific markers expressed on surfaceome with FDA-approved targeting. **e** Heatmap for TNBC specific markers expressed on surfaceome with FDA approved targeting

Many current therapeutics for breast cancer target cancer cells through cell surface expression of protein receptors: ER, PR, HER2. These therapeutics have yielded varying success in targeting luminal and HER2 + populations. However, breast cancer treatment for the most biologically proliferative subtype, TNBC, also has the least targeted treatment options available [56]. Unsurprisingly, there is a prominent academic and pharmaceutical effort to abate this gap in targeted treatment, highlighted by hundreds of investigational targeted therapies across disease subtypes. A compiled list of FDA-approved target molecules can be found in Supplementary Table 6. Individual cell clusters were analyzed to identify potential therapeutic. This assay allows more informed prediction of targeting and therapeutic response in cells for which the global expression of both 1) a target gene of interest and 2) genes involved in the inhibition pathway of this target exceeds a significant threshold. Figure 7 is a series of violin plots outlining the expression of common targets for disease and the pathways of inhibitions. The heatmaps were separated by subtype where Fig. 7a, b, and c represent Luminal, HER2 + , and TNBC, respectively. Most targets were present in a population of cells for their designated disease subtype. Expression of the target with a pathway of inhibition was compared to predict response to therapy. For example, Trop2 and Top1 are the target and pathway for Sacituzumab Govitecan-hziy, one of the only targeted therapeutics in TNBC.

Once gene expression has been incorporated to predict cellular response to known targets, extending this analysis for novel targets to breast cancer can provide new treatment options. To do this, a comprehensive vector of genes known to produce targetable proteins is overlaid with the gene expression matrix from the merged atlas dataset. Through this method, gene expression of both small molecule targets in the cellular cytosol, and gene expression of proteins expressed on cellular surfaces are selected. The generated highlights were then parsed for relevance in BC disease treatment. This includes conducting an extensive literature review to contrast these findings with ongoing studies across cancer types. Unsurprisingly, high concordance is observed of novel genes in BC across other cancer types. This can be due to fundamental similarities in cancer progression, such as VEGF dependency and immune evasion capacity. To highlight the potential efficacy of this pipeline in clinical care, our global datasets were selected for HER2 + and TNBC cell clusters. Using rownames() and selecting for significantly expressed genes (pct. > 0.25), two comprehensive gene vector sets expressed by HER2 + and TNBC subpopulations were generated. These vectors were then overlaid to identify genes unique to a disease. The concluding vectors provide a method of resolving genes with more isolated expression to the cancer cell types of interest. The filtered gene set is then intersected with the known surfaceome. The intersecting genes were also compared to a database of drug targets of FDA-approved therapeutics. The heatmaps in Fig. 7d and e are the potential druggable targets in HER2 + and TNBC cell populations, respectively. Unique gene expression for each population subset is observed. In the HER2 + population, gene expression of markers currently under investigation as potential targeted therapies were identified: *FGFR4* [57], *ITGB1* [58], and *ERBB2* [59]. The heatmap also highlights genes previously characterized within breast cancer, however not in the context of a potential therapeutic target, for example, *TUBB* [60]. For TNBC, this analysis proves to be especially useful, as targeted therapeutics for this subtype are limited. There is also a significant expression of key markers that target specific cell subclusters or the generic TNBC population. Some features of interest were identified in literature, for example, gene *PDE4B* codes for cyclic nucleotides involved in signal transduction in the hydrolysis of cAMP [61]. The protein translated from this gene is a target of AN2728, a PDE inhibitor. *COL1A1* is a published biomarker and potential therapeutic target of ER + cancers. However, it also has significant gene expression on the TNBC BT-549 cell line, which supports the idea of repurposing current treatment options for niched patient types.

The data were merged with respective disease subtypes of cell line clusters to highlight the efficacy of the BSCLA cell lines in predicting therapeutic efficacy in tumors. For example, high concordance is observed between cell lines and respectively subtyped BC primary tumor cells in the expression of markers such as *ESR1* and *ERBB2*, but also observed for less characterized targets such as *PARP1*, *CDK4*, *CDK6*, and *MTOR*. Results from the tissue dataset further validate the efficacy of using these model systems to define potential therapeutic avenues in patient disease when characterized with high-resolution assays. For gene markers in HER2 breast cancers, possible targeting of individual clusters with *PLAT*, *ICAM1*, and *FGA* is observed. Similarly, for gene markers in TNBC, possible targeting through genes expressed across multiple populations include *FN1*, *PLAU*, and *PDGFRB*. The heatmap also highlights genes specific to subclusters, including *VWF*, *FLT1*, and *RAMP3* in the Mesenchymal-5 cluster.

To investigate gene-level effects on the therapeutic interventions, two cell lines were treated for HDAC6 inhibition, identified from a genome wide RNAi screen. MDA-MB-453 was predicted to respond to this intervention with higher sensitivity than MDA-MB-436 [62]. Supplementary Fig. 7 highlights the variability in expression between MDA-MB-453 and MDA-MB-436 cell lines pre and post HDAC6 inhibition. This altered gene expression predicts efficient targeting and inhibition in the MDA-MB-453 population and the limited effect on MDA-MB-436. For example, in comparing network plots generated to compare treated and un-treated gene expression vectors for each cell line, the MDA-MB-453 analysis reveals pathways involved in morphogenesis, development, and apoptotic process attributed to increased gene expression markers including *G6PD*, *HSPB1*, *KRT8*, and *KRT19*. The MDA-MB-436 comparison yields fewer fundamental gene changes such as genes involved in nuclear speck and response to light, both pathways altered during microscopy events. Findings support this interpretation, with Cardillo et al. concluding MDA-MB-453 to be sensitive to sacituzumab govitecan [63]. This analysis does not factor in delivery method and transportation to cells. Still, it is instead focused on demonstrating the use of the BSCLA for predicting response to therapeutic once directed at the cancer cells.

## Discussion

By utilizing scRNAseq data for gene expression analyses in conjunction with high throughput single-cell functional predictions, we have developed a resource for distinct population identification and validation. Through publicly available scRNAseq data analysis tools, we have demonstrated the prediction of functional clusters within complex cell lines. This highlights the need for further investigation of high levels of intrinsic heterogeneity that must be considered when interpreting results. This heterogeneity may confound conclusions of past and current studies using both model systems and primary BC tissue. Cell lines are used across disciplines and have unknown levels of subpopulation variability, which can alter the ability to draw definitive conclusions from in vitro studies. In the case of therapeutic development, in vitro, human cell lines have been critical systems for predicting both the efficacy and toxicity of drugs.

In this work, we have generated high-resolution data characterizing model cell lines for the landscape of breast cancer disease. ScRNAseq data from 26 primary tumors is merged with the cell line dataset to inform this analysis further. The BSCLA has provided a framework for understanding the preliminary model systems we use and their relevance toward disease subtypes. We demonstrate the potential benefit in identifying heterogeneity of response in experimentation and an avenue of data generation potentially valuable for novel target identification through this improved characterization. To date, this provides the most comprehensive single-cell gene-level annotation of BC cell lines and may also include data synergistic for additional pathologies as more cell lines are characterized across other cancers in the future.

One of our primary methods of organizing this data analysis was identifying populational divergence on a pseudo-bulk level. The phylogenetic tree organized our clustered dataset by computing distance relationships. Genes driving divergence provide critical annotations of cell line populations. We identify published and novel gene markers highlighting epithelial and mesenchymal cell types as the primary nodes of differentiation. Next, we leveraged the high-resolution data across disease subtypes to generate gene vectors specific to each cell line and even subpopulations within cell lines. These molecular features of cell line subpopulations were previously unidentifiable through conventional bulk-RNA sequencing. Cell line gene vectors contribute to a deeper understanding of cellular function and provide a framework for modeling patient disease through a cocktail of cell line populations. The gene vector defining each cell line can be overlaid with patient scRNAseq data to identify a combination of model lines creating cell populations representative of individual disease. For example, SKBr3 and T47D cell lines can better represent different epithelial clusters, whereas BT549 represents many of the mesenchymal groups better.

We also evaluated our dataset for cell populations similar to CSCs or potential for stemness. We identify cell lines with a subpopulation of interest with stemness potential by overlaying published CSC marker expression across subpopulations. The sorted cell lines provide a whole population ranking of cell lines based on their CSC potential. The findings from this analysis support our ranking system from published markers. In contrast to graph-based trajectory inference methods such as Monocle, Wishbone, or Diffusion Pseudotime, several methods have been introduced leveraging estimates of information entropy surrounding gene expression profiles as a proxy measure of differentiation. Approaches, such as SLICE, SCENT, and Markov Chain Entropy, utilize Shannon Entropy as a means to quantify the degree of gene–gene interactions and pathway pruning occurring in each individual single-cell [64]. These recent studies have all demonstrated that single-cell gene expression entropy is thought to inversely correlate with the degree of differentiation (i.e., stemness) in both normal and cancer tissues [24]. It is believed that as cells differentiate, transcriptional regulatory programs prune away signaling pathways unnecessary to a given cell's committed fate, which is reflected in measurements of information entropy between presumably interacting gene pairs. Scores are calculated for each cell independently for one another, eliminating the possibility of trajectory bias from inaccurate or incomplete clustering required for the majority of graph-based methods [65]. Following entropy calculations, its impact on gene expression is estimated by conducting multiple parallel spearman rank correlations, followed by correction for multiple testing. Cells can then be aligned in order of increasing or decreasing entropy, and spline-curves fit to gene expression to examine changing expression dynamics.

We observed a high correlation between published CSC markers and entropy scoring. By overlaying differential genes and sorting by entropy score, we generated gene vectors for our population as potential pathways for CSC identification, including *ITGA6*, *CD44*, and *ITGB1*. Next, we identify markers from each cell line with concordance to entropy score to generate unique CSC genes within each cell line. Intriguingly, we notice discordant CSC gene expression versus entropy scores when comparing disease subtypes. Luminal cell lines have an inverted relationship between key genes and entropy score than TNBC cell lines, supporting the CSC model in disease recurrence. Canonical markers of cancer stemness share a positive Spearman correlation with entropy in TNBC cell lines. We believe this agreement highlights the increased prevalence of the CSC cell population in TNBC, providing an improved opportunity for cancer cell survival and recurrence post-therapy.

As a byproduct of subtyping BC disease through cell surface expression, there is an inevitable paradox in classifying disease. With improved techniques in characterizing cell populations, grouping cells by protein expression despite higher resolution metrics provides in some cases inconsistent annotation. Using the BSCLA, we can reannotate cell line disease subtypes and filter primary tumor cells by single-cell gene expression. When doing so, we validate the protein level cell line classifications for 9 of 13 unique cell lines. We observe heterogenous ERBB2 expression between clusters of cell lines within SUM190 and MDA-MB-453. This variability in expression indicates the potential of cell lines to have variable disease subtypes. This finding reflects our evolving understanding of the plasticity of breast cancer tumors, as summarized by Yeo and colleagues in identifying separate disease entities within individual patient tumors [66]. While we observe concordance with many of the disease subtype annotations from literature, there are some interesting variabilities in subtyping binning. For example, data from patient sample CID4067 is annotated as ER + . However, this sample source publication has significantly high *ERBB2* gene expression compared to the dataset, thereby evaluating it as HER2 + in this analysis. These variabilities can be attributed to publications using patient diagnostic information to bin subtypes rather than gene expression of the cells composing the data.

With the reclassified cell lines, we pooled cells by disease category to define gene divergences. Some cell line subpopulations such as SUM190 and MDA-MB-453 were classified into different Seurat objects depending on *ERBB2* expression. We observe unique gene expression representing these populations, particularly increased expression of genes sharing the *ERBB2* amplicon. *GRB7* has previously been associated with overamplification with *ERBB2*. These consistent modules support androgen receptor (AR) signaling as a driving force for tumorigenesis, thereby also opportuning therapeutic cocktails for HER2 + and AR targeting. We also observe marker differences controversial to published correlations such as *TLK1,* which was believed to be most amplified in Luminal B breast cancer [67]. When evaluating HER2 + populations by GSEA, a large percent of the population is activating genes in the biological process pathway, which are typically reserved for genes whose bioproduct is unknown. Some genes identified by the network plot highlight S100A gene set activation across the HER2 + dataset. For example, genes such as *S100A8* have been associated with malignancy and activation in HER2 + [48].

Sample sources with confirmed overexpression of HER2 were merged and reclustered for deeper analysis. The 3 sample sources, HCC1954, BT-474, and CID3921, generated 5 functional subclusters. Conserved expression of markers such as *S100A10* indicates metastatic potential, paralleling known biology of disease aggressiveness within HER2 expressing breast cancer [68]. DGEA between clusters yields unique inferences regarding the functionality of the larger partitions observed by the UMAP plots. For example, gene expression of *LCN2* in HER2-0 indicates mesenchymal phenotype, as described by Lu et al. [69]. Similarly, gene expression of collagen markers such as *COL1A2* infer HER2-4 to have fibroblast invasion [70]. The presence of CID3921 source cells across functional clusters indicates the relevance of cell lines in modeling tumor tissue. It further highlights the need for deep model characterization as one HER2 + cell line would not reflect the heterogeneity of the patient sample as well as both did.

To further investigate and validate functional inferences made from scRNASeq data, MDA-MD-453 cells were processed through fluorescent microscopy assays measuring the expression of ERBB2. Results of measured gene expression by scRNASeq, surface marker expression by high throughput microfluidic imaging, and traditional microscopy are concordant in illustrating the variability of expression. Through protein level characterization of the MDA-MB-453 cell line, we are able to validate predicted gene level heterogeneity inferences made. The heterogeneity observed here reflects previously published variability in the cell line’s subtype annotation [11]. We believe the data presented here adds to the transcriptomic inferences made in the paper in two ways: 1) by validating gene-protein association and 2) by providing a framework for functional testing of cell samples that matches the throughput of current scRNASeq. Overall, the heterogeneity in expression of ERBB2 found from single-cell RNA data translated to surface marker expression heterogeneity. These findings further confirm the variability within this cell line that we identified in our literature review. We did observe a disproportionate number of cells positive for ERBB2 expression between gene and protein assays and believe this difference is reflected by a few factors. One potential factor is discordant gene and protein expression due to translational pathway interference between gene expression and cell surface marker development, previously indicated by Wegler et al. [71]. Another potential factor is cell culture states at the time of sample processing, where ERBB2 expressing cell representation may fluctuate over culture timepoints, previously indicated by Sato et al. when running lineage tracking on HeLa cells [72]. Another factor potentially addressing this discrepancy is assay thresholding variability between gene and protein analysis. For example, within the microfluidic system, ERBB2-negative identified cells have an expression of ERBB2; however, the selection of expressing versus overexpressing cells for positive identification may affect our analysis. Overall, findings support known but uncharacterized heterogeneity within cancer cell lines. Previous work by TCGA (The Cancer Genome Atlas), CCLE (Cancer Cell Line Encyclopedia), and many independent researchers have established a deep foundational understanding of cell lines on the bulk-Seq and protein levels. With the advent of high-throughput and high-resolution assays, there is an unmet need to apply these assays to model systems such as cell lines. We believe the work presented here addresses some of these unmet needs and provides further support for continued deeper investigation into characterizing model systems in future works.

While most of the analysis focused on validating and resolving the current understanding of BC cell lines, we believe this high-resolution data generation can also be leveraged for novel marker and therapeutic identification across the dataset. This is illustrated by the expression patterns of therapeutic targets and pathways of inhibition in current clinical treatment options. Epithelial-11 is an example of a subpopulation that may be targeted successfully due to HER2 expression predicted by *ERBB2* expression. However, a treatment option that acts upon CDK6 activity could be less effective due to the low expression of the *CDK6* gene. To identify potential novel treatment targets, we overlayed significantly expressed genes in TNBC and HER2 + populations, with genes known to translate into surface markers. When intersecting this dataset with FDA-approved targets, we generate over 100 potential targets in HER2 + and TNBC cell population for cell lines and primary tumor clusters. Within HER2 + gene markers of interest, we generate hits novel genes predicted to be expressed on the surfaceome of HER2 + clusters. These serve as a means of targeting specific populations that have evaded current therapy. For example, the *PLAT* gene is expressed primarily in three clusters of primary tumor samples, including Epithelial-3, Epithelial-6, and Epithelial-9. These clusters exhibit lower expression of *ERBB2* compared to other epithelial clusters and cell line clusters and serve as candidate cell populations for alternative targeting. In the TNBC analysis, some of the genes are highly annotated markers for TNBC disease, including *ITGB1* and *CD44*, where both genes have been identified as prognosis makers [73, 74]. Similarly, some other genes identified have shown early-stage benefit as a target for TNBC treatment, such as *EGFR* [75]. The analysis also reveals novel signatures not as prevalent in current publicly available data. For example, we identify *M6PR* as a potential novel cell surface marker and targetable molecule. This analysis pipeline assumes that observed gene expression translates to targetable protein expression, which can be further refined and validated through future data generation on platforms such as CITE-seq.

While this scRNAseq analysis generated abundant data and insights about BC cell lines, it followed with some limitations. The work presented here analyzes differential gene expression within breast cancer cell lines to predict subpopulations. Inferences on subpopulation function leverage unique genes to each population and published work on functional experimentation of specific genes. Another limitation is the observed heterogeneity between culture samples of MCF-7, most of the data is limited to a singular timepoint for a cell line cultured from a particular lab source. While this data adds a level of understanding about culture heterogeneity, it also further sheds light on the sensitivity of these characterizations to timepoints and culture conditions. Similarly, generating high-resolution data at multiple time points of culture across cell lines can further support stemness investigations. Additionally, with scRNAseq data, we attempt to interpret heterogeneity and functional values of cell types. However, gene and protein expression have been shown to vary, and therefore the predictive statements would be supported with future validation experimentation [76]. Assays such as CITE-seq and single-cell copy number variation (CNV) can clarify phenotypic and genomic sources of heterogeneity, respectively.

## Conclusion

As the standard of oncology treatment moves toward targeted therapies, our understanding of model systems used as the first line of testing needs to be improved through higher resolution characterization. Further scRNAseq investigations paired with phenotypic observations can provide the required level of deep insight into cell populations used for these types of critical studies. Here we have presented a comprehensive but preliminary investigation into the presence and roles of cellular diversity within cell lines and primary tumors. Previous work has provided high-resolution data on patient samples or bulk level characterization of model cell lines [38, 77]. This atlas is a comprehensive single-cell breast cancer cell line dataset, unique in its contribution by providing a tool for cell line characterization and selection which we believe will improve efficiency and accuracy of legacy research. In addition to the cell line data, we overlay model data with a breast tumor atlas, providing further understanding towards cell line functionality and representation of tumor heterogeneity. Furthermore, we extend our analysis to understanding gene expression alterations post novel therapeutic treatment, while also indicating capacity to predict efficacy. Lastly, we leverage novel and custom-engineered analysis pipelines serving as proof-of-concepts for unsupervised cell annotation and cancer stem cell prediction.

We believe this dataset should encourage researchers to further develop higher resolution data points for patient cases and the model systems we use to understand them. Through higher resolution data generation, we resolved subtype heterogeneity, identified subclusters across our dataset with higher probability likelihood of stemness, elucidated sub passage and lab effects on transcriptome with MCF-7, developed deep gene-level predictive values for current treatment options in breast cancer, overlayed gene expression of resistive cancer types with FDA approved targets on surfaceome to generate novel treatment targets. All the metrics we generate function as a predictive tool in the complex landscape of breast cancer. As such, our results need validation on a protein and DNA level. With the advent and development of single-cell assays, we believe this paper provides support for further cumulative effort in characterizing the heterogeneity in breast cancer. Through this deeper analysis, we show there are substantial and direct implications on how we view disease and clinical decision-making. Our BSCLA atlas of 75,409 cells from 13 distinct cell lines and 26 primary tumors defines and categorizes heterogeneous subpopulations across disease states. We envision the incorporation of this atlas across breast cancer investigations. Therefore, we provide foundational investigations into the dataset, including resolving subtyping through higher resolution data generation, predicting novel therapeutic targets, and generating deeper pathway analyses to define population divergence.

## Supplementary Information

Below is the link to the electronic supplementary material.

Supplementary file1 (PDF 466 KB)

Supplementary file2 (PDF 939 KB)

Supplementary file3 (PDF 256 KB)

Supplementary file4 (PDF 479 KB)

Supplementary file5 (PDF 2743 KB)

Supplementary file6 (PDF 582 KB)

Supplementary file7 (PDF 722 KB)

Supplementary Tables (XLSX 37.8 KB)

## Data Availability

The datasets generated during and/or analysed during the current study are available in the Gene Expression Omnibus repository, ID GSE182694 (Data generated in this investigation) and GSE176078 (Previously published data by Wu et al.).
