# Supplementary figures and images for: The Breast Cancer Single-Cell Atlas: Defining cellular heterogeneity within model cell lines and primary tumors to inform disease subtype, stemness, and treatment options
Source: Cell Oncol (Dordr). 2023 Jan 4;46(3):603–28. doi: 10.1007/s13402-022-00765-7 (PMC10205851; doi:10.1007/s13402-022-00765-7)

**a.****Cell Type Representation in Generated Clusters**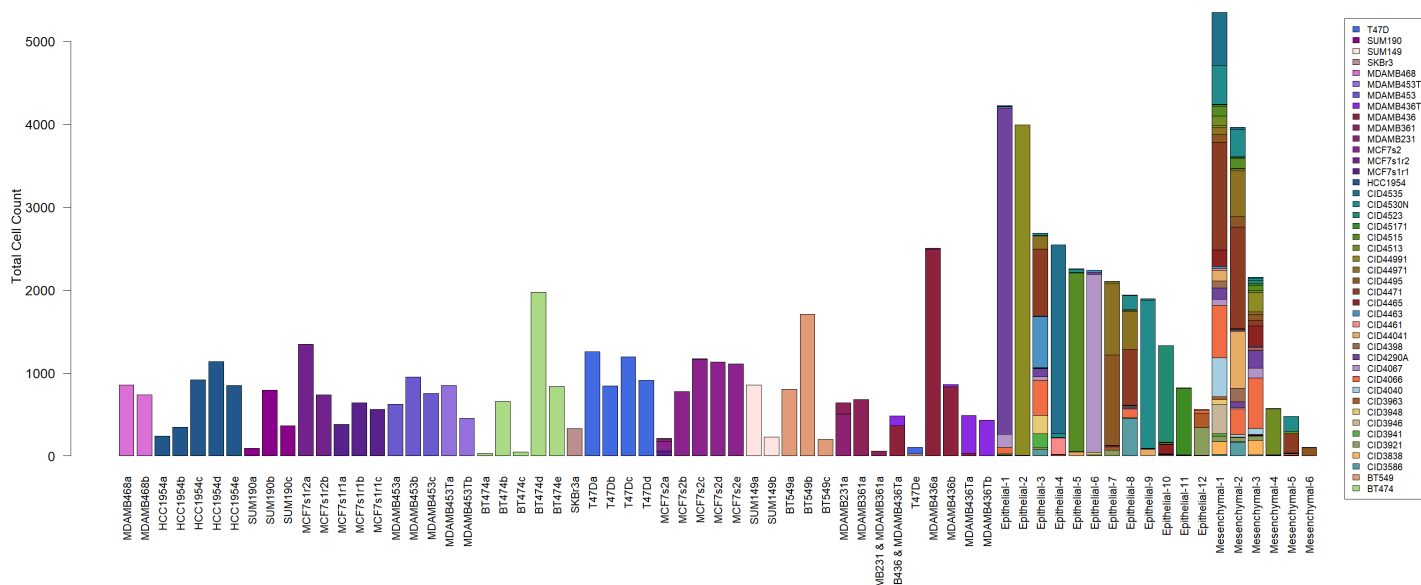**b.**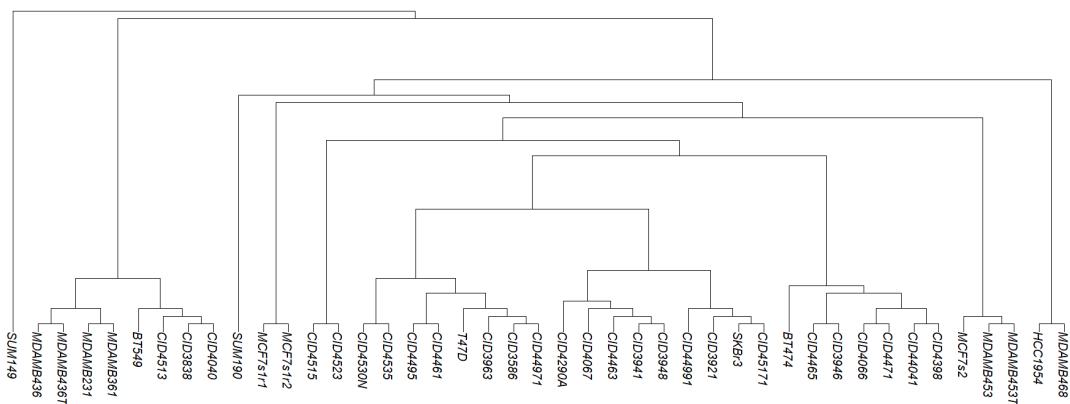

Supplement: Supplementary file 1 — Supplementary file1 (PDF 466 KB) [file 13402_2022_765_MOESM1_ESM.pdf]

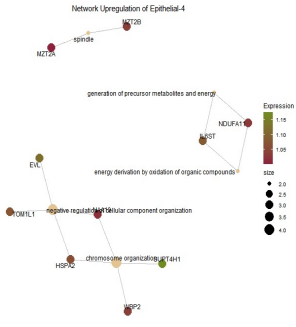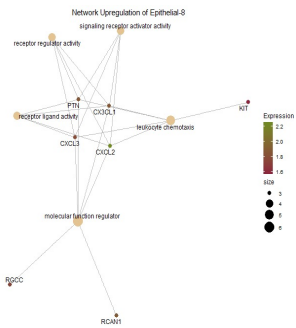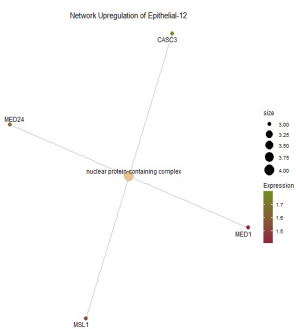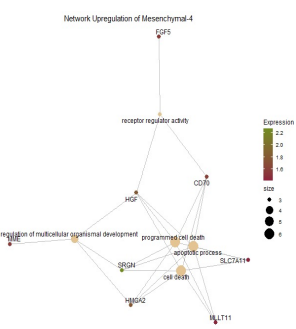

Supplement: Supplementary file 2 — Supplementary file2 (PDF 939 KB) [file 13402_2022_765_MOESM2_ESM.pdf]

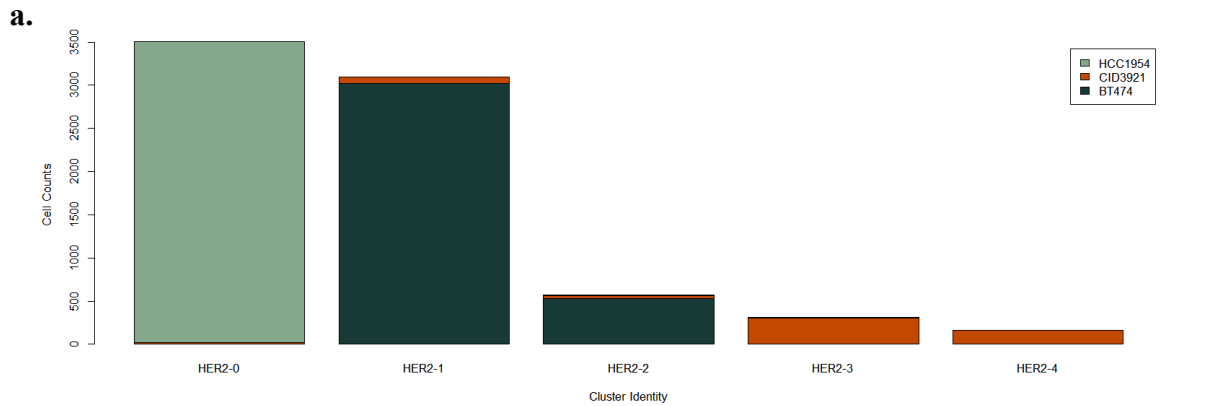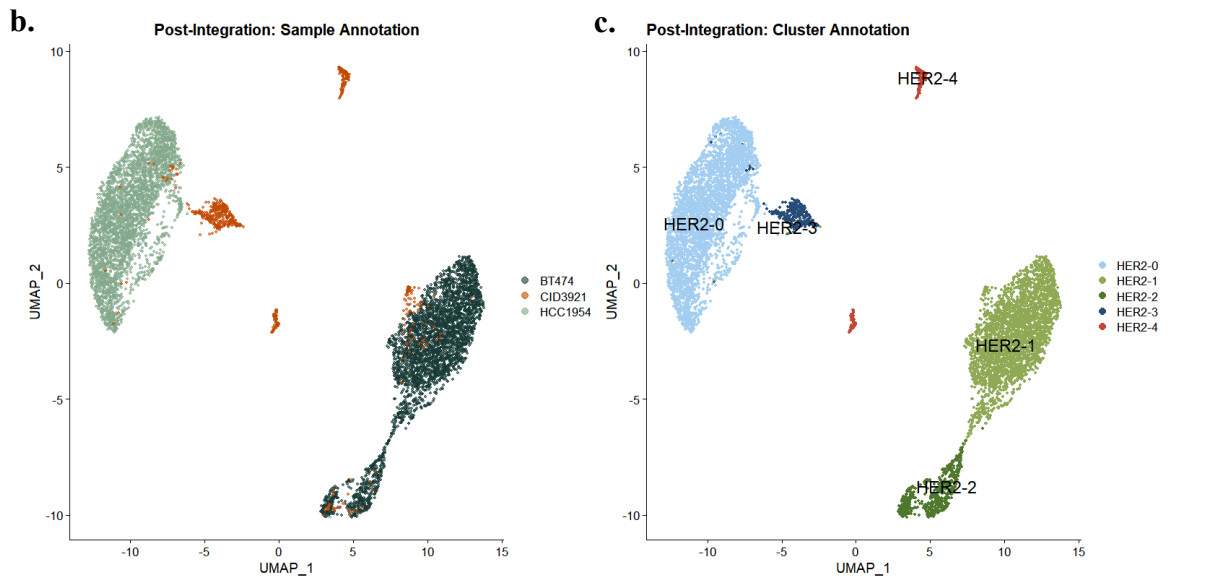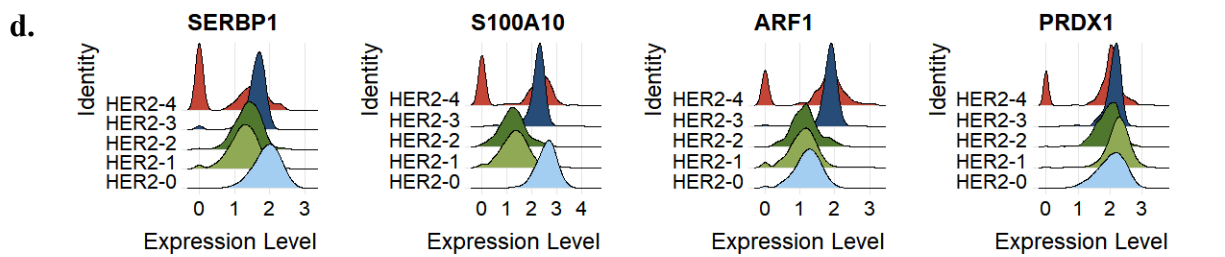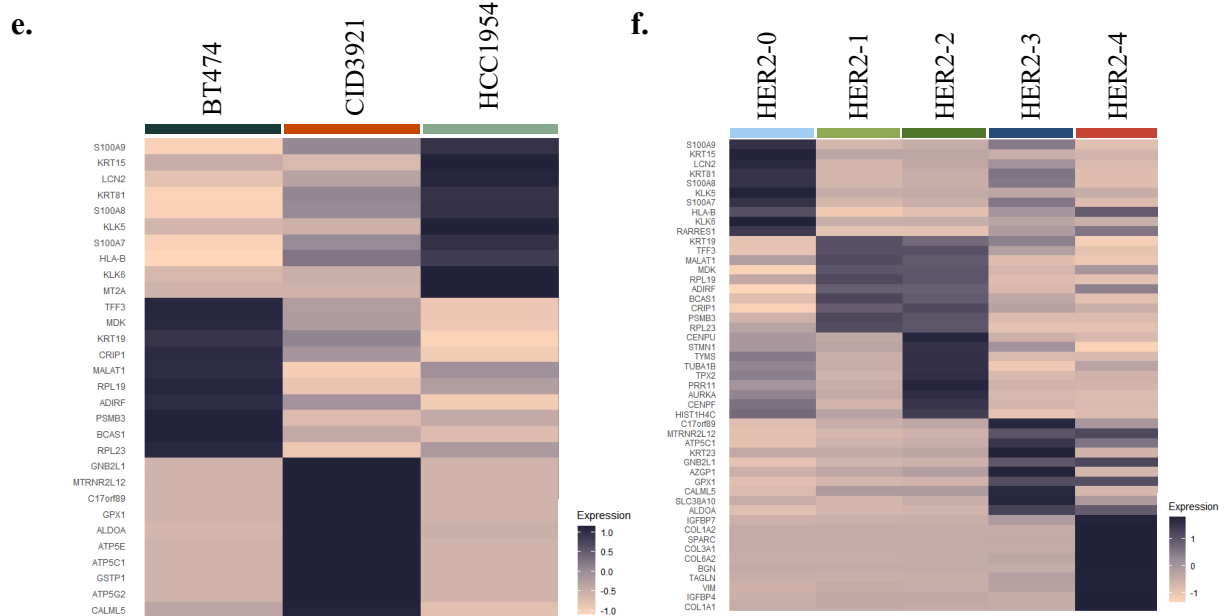

Supplement: Supplementary file 3 — Supplementary file3 (PDF 256 KB) [file 13402_2022_765_MOESM3_ESM.pdf]

## MDA-MB-436

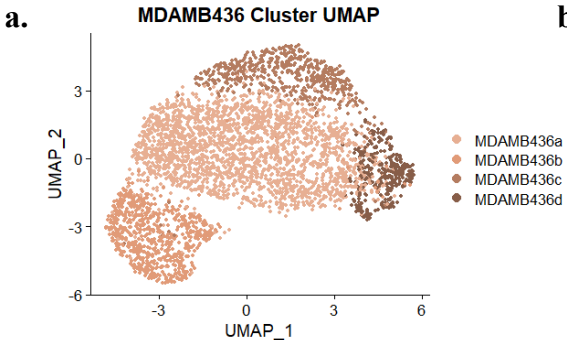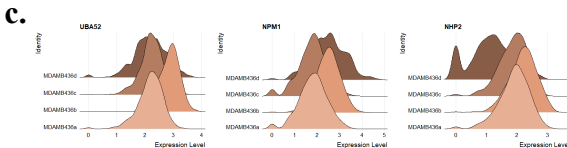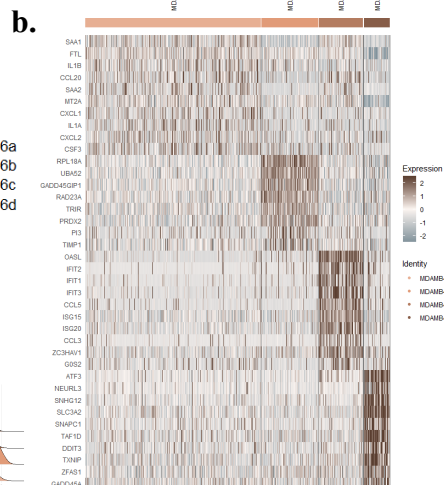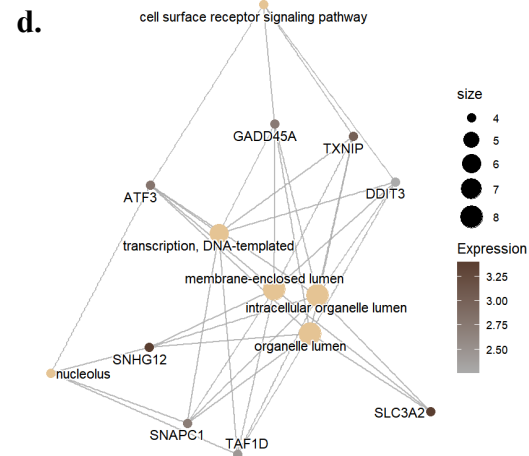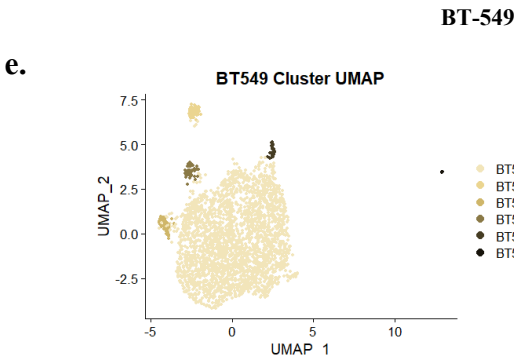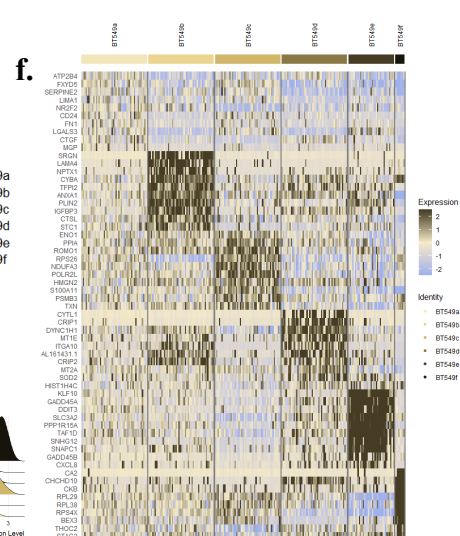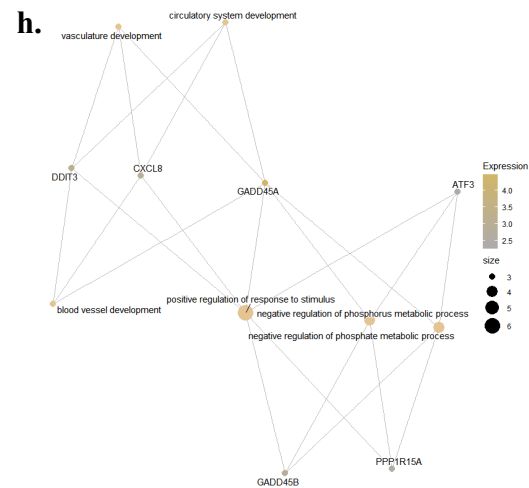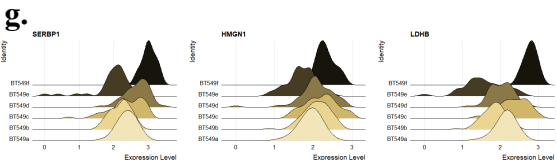

### MCF-7 INTER SAMPLE ANALYSIS

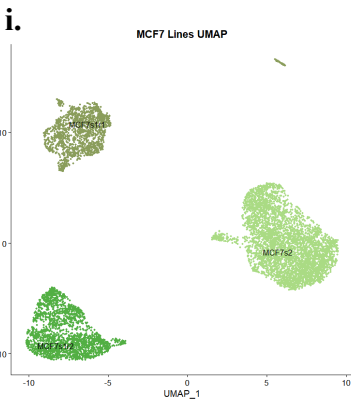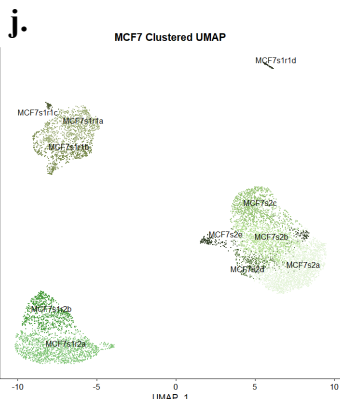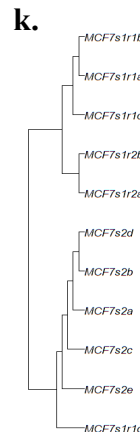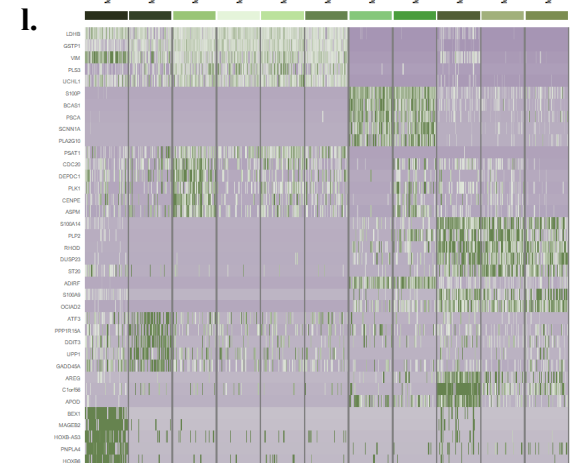

Supplement: Supplementary file 6 — Supplementary file6 (PDF 582 KB) [file 13402_2022_765_MOESM6_ESM.pdf]

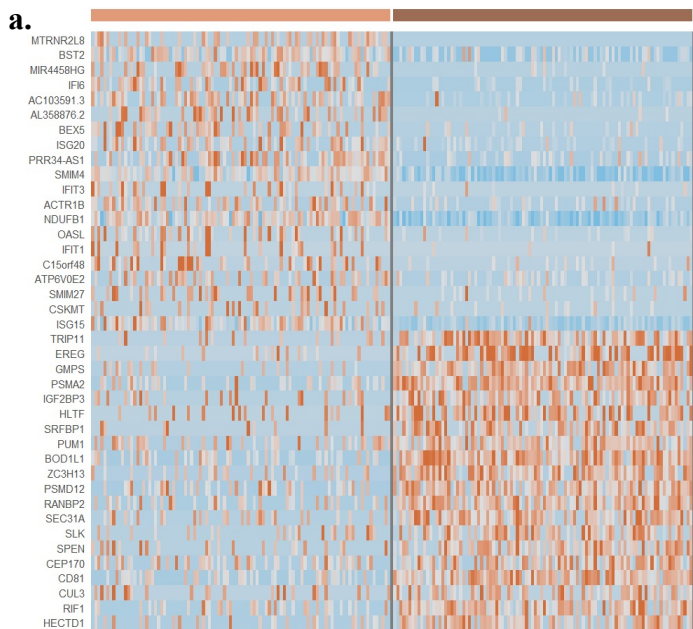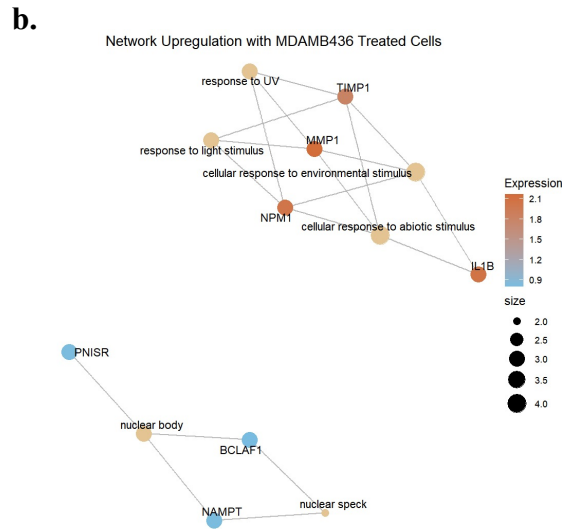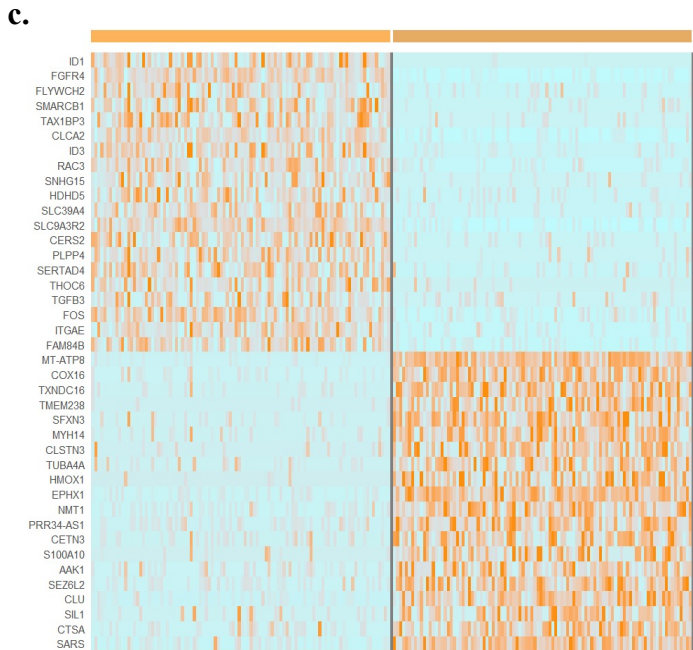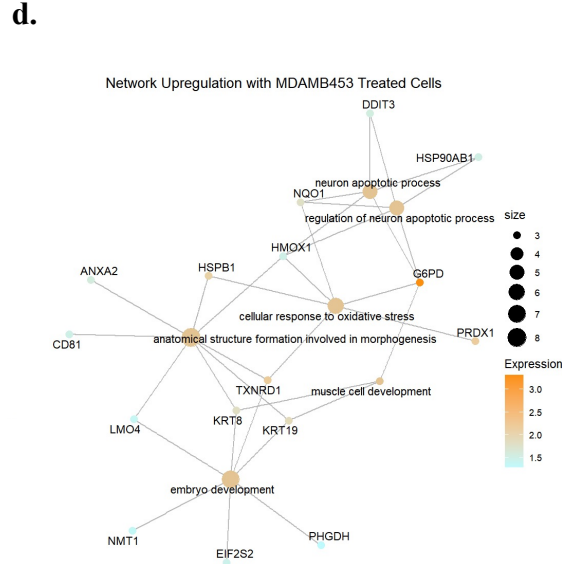

Supplement: Supplementary file 7 — Supplementary file7 (PDF 722 KB) [file 13402_2022_765_MOESM7_ESM.pdf]
