# Supplementary material for: The Breast Cancer Single-Cell Atlas: Defining cellular heterogeneity within model cell lines and primary tumors to inform disease subtype, stemness, and treatment options
Source: Cell Oncol (Dordr). 2023 Jan 4;46(3):603–28. doi: 10.1007/s13402-022-00765-7 (PMC10205851; doi:10.1007/s13402-022-00765-7)
Supplement: Supplementary file 4 — Supplementary file4 (PDF 479 KB) [file 13402_2022_765_MOESM4_ESM.pdf]

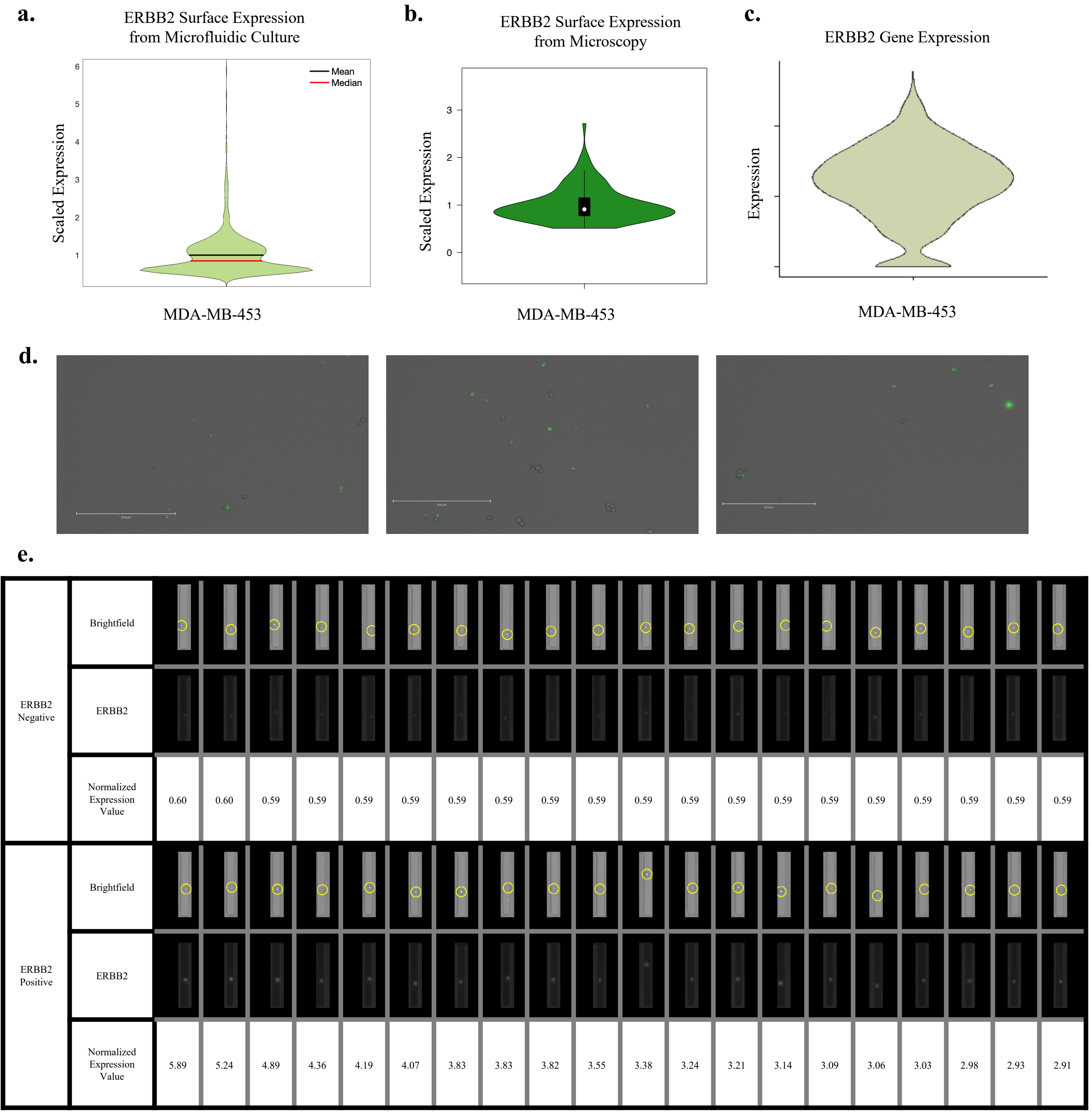
a.
ERBB2 Surface Expression from Microfluidic Culture
Scaled Expression
1
2
3
4
5
6
Mean
Median
MDA-MB-453
b.
ERBB2 Surface Expression from Microscopy
Scaled Expression
0
1
2
3
MDA-MB-453
c.
ERBB2 Gene Expression
Expression
MDA-MB-453
d.
e.
ERBB2 Negative
Brightfield
ERBB2
Normalized Expression Value
0.60 0.60 0.59 0.59 0.59 0.59 0.59 0.59 0.59 0.59 0.59 0.59 0.59 0.59 0.59 0.59 0.59 0.59 0.59 0.59
ERBB2 Positive
Brightfield
ERBB2
Normalized Expression Value
5.89 5.24 4.89 4.36 4.19 4.07 3.83 3.83 3.82 3.55 3.38 3.24 3.21 3.14 3.09 3.06 3.03 2.98 2.93 2.91
