# Supplementary material for: The Breast Cancer Single-Cell Atlas: Defining cellular heterogeneity within model cell lines and primary tumors to inform disease subtype, stemness, and treatment options
Source: Cell Oncol (Dordr). 2023 Jan 4;46(3):603–28. doi: 10.1007/s13402-022-00765-7 (PMC10205851; doi:10.1007/s13402-022-00765-7)
Supplement: Supplementary file 5 — Supplementary file5 (PDF 2743 KB) [file 13402_2022_765_MOESM5_ESM.pdf]

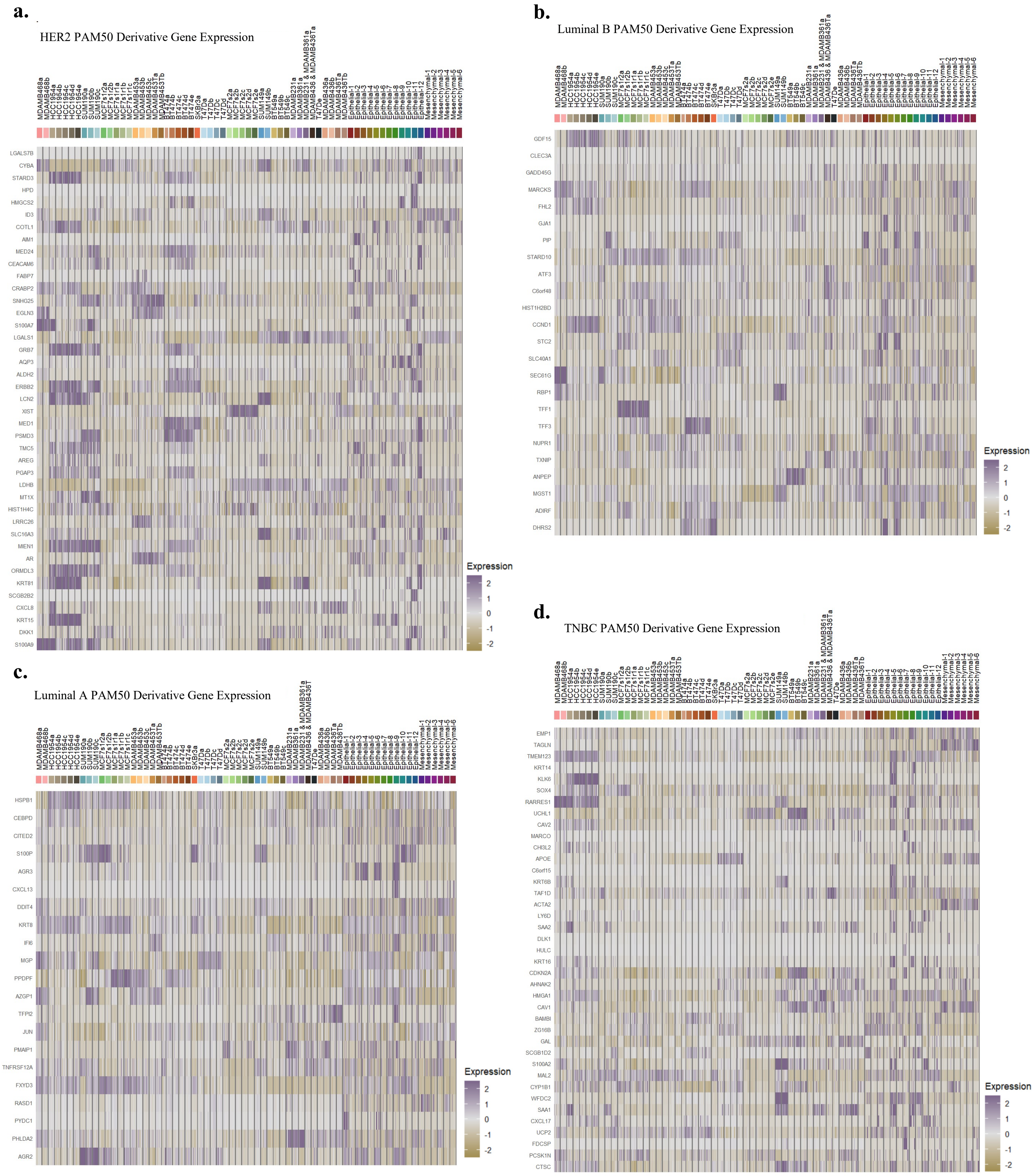
a.
HER2 PAM50 Derivative Gene Expression
LGALS7B
CYBA
STARD3
HPD
HMGCS2
ID3
COTL1
AIM1
MED24
CEACAM6
FABP7
CRABP2
SNHG25
EGLN3
S100A7
LGALS1
GRB7
AQP3
ALDH2
ERBB2
LCN2
XIST
MED1
PSMD3
TMC5
AREG
PGAP3
LDHB
MT1X
HIST1H4C
LRRC26
SLC16A3
MIEN1
AR
ORMDL3
KRT81
SCGB2B2
CXCL8
KRT15
DKK1
S100A9
Expression
2
1
0
-1
-2
b.
Luminal B PAM50 Derivative Gene Expression
GDF15
CLEC3A
GADD45G
MARCKS
FHL2
GJA1
PIP
STARD10
ATF3
C6orf48
HIST1H2BD
CCND1
STC2
SLC40A1
SEC61G
RBP1
TFF1
TFF3
NUPR1
TXNIP
ANPEP
MGST1
ADIRF
DHRS2
Expression
2
1
0
-1
-2
c.
Luminal A PAM50 Derivative Gene Expression
HSPB1
CEBPD
CITED2
S100P
AGR3
CXCL13
DDIT4
KRT8
IFI6
MGP
PPDPF
AZGP1
TFPI2
JUN
PMAIP1
TNFRSF12A
FXYD3
RASD1
PYDC1
PHLDA2
AGR2
Expression
2
1
0
-1
-2
d.
TNBC PAM50 Derivative Gene Expression
EMP1
TAGLN
TMEM123
KRT14
KLK6
SOX4
RARRES1
UCHL1
CAV2
MARCO
CHI3L2
APOE
C6orf15
KRT6B
TAF1D
ACTA2
LY6D
SAA2
DLK1
HULC
KRT16
CDKN2A
AHNAK2
HMGA1
CAV1
BAMBI
ZG16B
GAL
SCGB1D2
S100A2
MAL2
CYP1B1
WFDC2
SAA1
CXCL17
UCP2
FDCSP
PCSK1N
CTSC
Expression
2
1
0
-1
-2
